# Multigeneration Chemistry in Secondary Organic Aerosol Formation from Nitrate Radical Oxidation of Isoprene

**DOI:** 10.1021/acsearthspacechem.4c00417

**Published:** 2025-01-27

**Authors:** Tianchang Xu, Masayuki Takeuchi, Jean C. Rivera-Rios, Nga L. Ng

**Affiliations:** † School of Chemical & Biomolecular Engineering, Georgia Institute of Technology, Atlanta, Georgia 30332, United States; ‡ School of Civil & Environmental Engineering, Georgia Institute of Technology, Atlanta, Georgia 30332, United States; § School of Earth & Atmospheric Sciences, Georgia Institute of Technology, Atlanta, Georgia 30332, United States

**Keywords:** isoprene, biogenic volatile organic compounds, secondary organic aerosol, SOA, multigeneration chemistry, nighttime oxidation, organic nitrate, volatility, aerosol yields

## Abstract

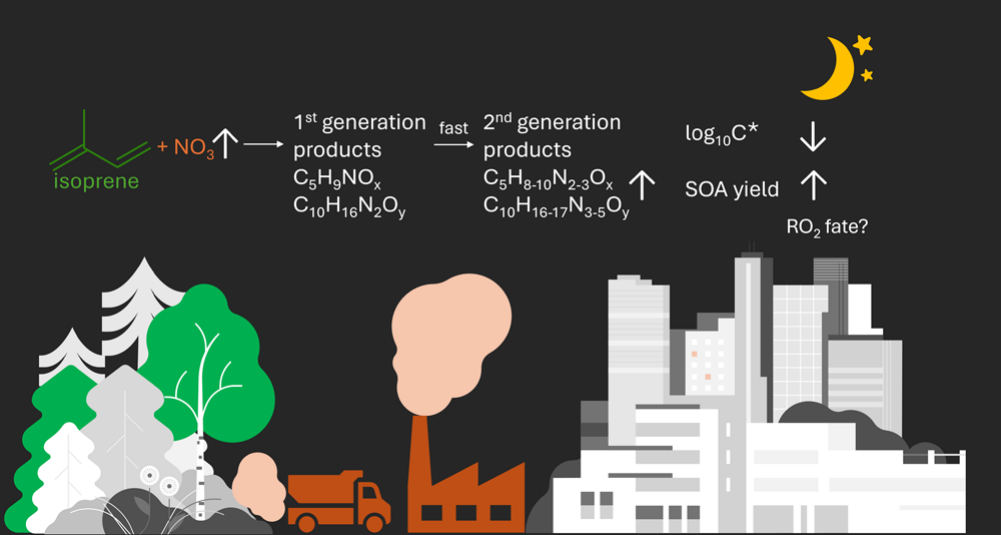

The nitrate radical (NO_3_) oxidation of isoprene is an important contributor to secondary organic aerosol (SOA). Isoprene has two double bonds which allow for multigeneration oxidation to occur. The effects of multigeneration chemistry on the gas- and particle-phase product distributions of the isoprene + NO_3_ system are not fully understood. In this study, we conduct chamber experiments by varying the ratio of N_2_O_5_ (precursor of NO_3_) to isoprene concentration from 1:1 to 14:1 to investigate the formation of products in both phases under different oxidation levels. Multigeneration chemistry leads to the formation of gas-phase products which then partition into particle phase depending on the product volatility; first-generation products (15–36% of total SOA) such as C_5_H_9_NO_5_ and C_10_H_16_N_2_O_9_ have volatility (log_10_C* = 1.0–3.0 using the partitioning method and log_10_C* = 2.6–4.5 using the formula method) 1–5 orders of magnitude higher than second-generation products (37–57% of total SOA, log_10_C* = −0.8–2.1 using the partitioning method and log_10_C* = −3.7–1.8 using the formula method) such as C_5_H_8,10_N_2_O_8_, C_5_H_9_N_3_O_10_, and C_10_H_17_N_3_O_13_. The fast reaction rate constants of first-generation products (estimated to be on the order of 10^–13^ cm^3^ molecules^–1^ s^–1^ at 295 K) and the lower volatility of second-generation products result in increased SOA yields when NO_3_ availability increases and multigeneration chemistry is enhanced. Specifically, an increase of up to 300% in SOA yield is observed when the N_2_O_5_/isoprene ratio increases from 1:1 to 3:1; from 5.7% (organic aerosol mass concentration, Δ*M*_o_ = 4.2 μg/m^3^) to 16.3% (Δ*M*_o_ = 11.9 μg/m^3^) when the reacted isoprene concentration is 25 ppb and from 3.1% (Δ*M*_o_ = 1.2 μg/m^3^) to 12.4% (Δ*M*_o_ = 5.4 μg/m^3^) when the reacted isoprene concentration is 15 ppb. The maximum SOA yield occurs when the N_2_O_5_/isoprene ratio is greater than or equal to 3:1 as a combined result of multigeneration chemistry and peroxy radicals (RO_2_) fate. We encourage future studies to consider both factors, which can vary under different laboratory and ambient conditions, when comparing SOA yields to better understand any differences observed. Our results highlight that multigeneration chemistry and the updated parameters including reaction rate constants and volatility distribution of products should be considered to enable a more comprehensive representation and prediction of SOA formation from NO_3_ oxidation of isoprene in atmospheric models.

## 1 Introduction

The oxidation of biogenic volatile organic compounds (VOCs) by ozone, hydroxyl (OH), and nitrate radical (NO_3_) contributes substantially to secondary organic aerosol (SOA) formation on a global scale.^1−4^ Among biogenic VOCs, isoprene has the largest emission of approximately 500–1000 Tg per year.^5,6^ Isoprene is primarily emitted during the day as a byproduct of photosynthesis. Areas including the southeastern United States (U.S.), tropical rain forests, and even remote marine environments are reported to have high isoprene emissions.^6,7^ Isoprene reacts rapidly with atmospheric oxidants forming species with reduced volatility that partition into the particle phase. Modeling studies indicate that the amount of isoprene-derived SOA is as high as ∼150 Tg per year globally.^8,9^ Isoprene can be efficiently oxidized by OH radical, leading to daytime SOA.^10−16^ Residual isoprene emitted in the late afternoon is dominantly oxidized by NO_3_ and ozone (O_3_), but can also react with OH radical to form SOA at night.^17−23^ Multiple field campaigns have observed a rapid decrease of isoprene and an increase of isoprene-derived SOA after sunset, demonstrating that nighttime chemistry is an important source of SOA formation.^19,21,24,25^

However, our understanding of the formation mechanism and composition of isoprene-derived SOA from NO_3_ oxidation at night or during the day under a heavy canopy is incomplete. Since isoprene has two double bonds, multigeneration products can form rapidly in the atmosphere. First-generation products are defined here as products formed when NO_3_ initially reacts with one of the two double bonds in isoprene, while second-generation products result from subsequent reactions involving the other unreacted double bond of first-generation products. While the importance of multigeneration NO_3_ oxidation of isoprene in SOA formation has been acknowledged in previous work, there is still a lack of detailed investigation and comprehensive understanding beyond the first-generation oxidation.^26−28^ Recently, Wu et al. evaluated the role of multigeneration chemistry in the isoprene + NO_3_ reaction by measuring gas-phase oxidation products while varying the NO_3_ to isoprene ratio.^29^ They proposed that multigeneration oxidation can form both monomers (C_5_ products) and dimers (C_10_ products) with different numbers of substituted nitrate groups: first-generation products tend to be monomers with one nitrogen atom (1N-monomers) and dimers with two nitrogen atoms (2N-dimers), whereas second-generation products tend to be higher nitrogen-containing monomers and dimers. As the NO_3_ level increases, more second-generation products are formed in the gas phase with the decay of first-generation products. However, the effects of multigeneration chemistry on SOA formation, including product composition in particle phase, volatility, and SOA yield, remain uncertain, which makes it challenging to accurately predict SOA formation in models. Therefore, more comprehensive, simultaneous measurements of chemical composition in both gas and particle phases are needed to better constrain the SOA formation mechanism from gas-particle partitioning of multigeneration products.

In this study, we investigate changes in the SOA formation pathways from the reaction of isoprene and NO_3_ under different oxidation levels. A series of laboratory chamber experiments are conducted with varying levels of NO_3_, allowing us to study SOA generation over a range of oxidation conditions. We utilize both gas- and particle-phase chemical composition measurements to determine the effects of multigeneration chemistry on SOA formation. In particular, we focus on the composition, volatility distribution, and SOA mass yield of isoprene + NO_3_ products from multigeneration chemistry.

## 2 Experimental Section

### 2.1 Chamber Setup

Experiments are conducted in the Georgia Tech Environmental Chamber facility housing two 12 m^3^ Teflon chambers at 22 °C and under dry conditions (<5% RH).^30^ All experiments listed in Table 1 use ammonium sulfate (0.015 M) as the seed aerosol. After seed injection, isoprene (99% Sigma-Aldrich) is injected into a glass bulb and then evaporated into the chamber by flowing zero air through the glass bulb. Then, the oxidant precursor, N_2_O_5_, is generated by mixing NO_2_ (485 ppm, Matheson) and O_3_ (250 ppm, Jelight 610) in a flow tube and injected into the chamber at a rate of 11 ppb/min.^30−33^ The flow rates of NO_2_ and O_3_ are controlled to minimize the concentration of O_3_ to ensure that the VOC is dominantly oxidized by NO_3_ (i.e., >98% of isoprene reacts with NO_3_ according to 0-D model). N_2_O_5_ establishes equilibrium with NO_2_ and NO_3_ in the chamber, initiating the NO_3_ oxidation of isoprene.

**Table 1:** Initial Precursor and Oxidant Conditions, Calculated SOA Mass Yield, Effective Formula of SOA Products, Organic Nitrates Formed, and Product Volatility Information in This Study

set	expt	VOC_0_ (ppb)^a^	N_2_O_5_/VOC_0_^b^	ΔVOC (ppb)	Δ*M*_o_ (μg/m^3^)	SOA yield (%)^c^	effective formula^d^	pON/OA	log_10_C*^e^
A	1	30.8 ± 2.5	1:1	27.3 ± 2.5	4.2 ± 0.3	5.7 ± 0.7	C_7.8_H_13.4_O_9.3_N_2.1_	0.70 ± 0.02	2.97 ± 0.12
	2	24.8 ± 2.3	2:1	24.8 ± 2.3	7.5 ± 0.6	10.7 ± 1.3	C_7.8_H_13.3_O_9.7_N_2.2_	0.74 ± 0.02	2.87 ± 0.11
	3	25.0 ± 2.3	3:1	25.0 ± 2.3	10.3 ± 0.5	15.2 ± 1.5	C_7.9_H_13.3_O_9.9_N_2.3_	0.73 ± 0.02	2.85 ± 0.11
	4	24.3 ± 2.3	5:1	24.3 ± 2.3	10.9 ± 0.3	16.0 ± 1.6	C_7.9_H_13.1_O_10.0_N_2.4_	0.73 ± 0.02	2.92 ± 0.12
	5	25.9 ± 2.4	14:1	25.9 ± 2.4	11.9 ± 0.6	16.3 ± 1.8	C_7.7_H_12.6_O_10.1_N_2.4_	0.78 ± 0.03	2.95 ± 0.12
B	1	20.9 ± 1.1	1:1	14.0 ± 0.7	1.2 ± 0.2	3.1 ± 0.8	C_7.4_H_12.6_O_8.8_N_1.9_	0.84 ± 0.05	2.48 ± 0.10
	2	14.4 ± 0.7	2:1	14.4 ± 0.7	2.3 ± 0.1	5.7 ± 0.6	C_7.5_H_12.7_O_9.6_N_2.2_	0.85 ± 0.04	2.44 ± 0.10
	3	15.0 ± 0.8	3:1	15.0 ± 0.8	4.5 ± 0.1	10.6 ± 1.0	C_7.3_H_12.4_O_9.6_N_2.2_	0.90 ± 0.02	2.45 ± 0.10
	4	14.6 ± 0.7	5:1	14.6 ± 0.7	4.9 ± 0.1	12.0 ± 1.3	C_7.4_H_12.5_O_9.8_N_2.3_	0.87 ± 0.02	2.66 ± 0.11
	5	15.3 ± 0.8	14:1	15.3 ± 0.8	5.4 ± 0.1	12.4 ± 1.0	C_7.5_H_12.4_O_10.2_N_2.5_	0.88 ± 0.05	2.79 ± 0.11

a VOC uncertainty is calculated as one standard deviation from the gas chromatograph-flame ionization detector (GC-FID) calibration results.

b N_2_O_5_ concentration is calculated using a 0-D box model with known concentrations and flow rates of NO_2_ and O_3_ as inputs.

c Mass yield is calculated using particle wall loss corrected scanning mobility particle sizer (SMPS) data at peak SOA mass concentration. Uncertainty is calculated as the standard deviation of mass concentration during the peak SOA mass concentration period.

d Effective formula from FIGAERO-CIMS measurements at the peak SOA mass concentration cycle.

e The log_10_C* values correspond to the weighted average of log_10_C* of products that are present in both gas and particle phases (partitioning method), instead of SOA volatility, as described in Section 2.3.

Two sets of experiments with different amounts of reacted isoprene (ΔVOC) are conducted (set A with 15 ppb ΔVOC and set B with 25 ppb ΔVOC). We adjust the duration of the N_2_O_5_ injection to vary N_2_O_5_ concentration to initial VOC concentration ratio (N_2_O_5_/VOC) between 1:1 and 14:1 to systematically investigate multigeneration chemistry in isoprene SOA formation. In each set of experiments, we inject the same amount of isoprene except for experiments with the lowest N_2_O_5_/VOC ratio (experiments A1 and B1). For these two experiments, we increase the initial isoprene concentration by 5 ppb to keep the ΔVOC consistent with other experiments that have higher N_2_O_5_/VOC ratios.

### 2.2 Instrumentation

Isoprene concentration is measured with a gas chromatograph-flame ionization detector (GC-FID, Agilent 7890 A) with a PLOT-Q column (Agilent). Particle number and volume concentrations are measured by a scanning mobility particle sizer (SMPS), which is composed of a differential mobility analyzer (TSI 3080) and a condensation particle counter (TSI 3775). A high-resolution time-of-flight aerosol mass spectrometer (HR-ToF-AMS, Aerodyne Research Inc.) is used to measure the mass concentrations of submicron nonrefractory aerosol, including organics, nitrate, sulfate, ammonium, and chloride.^34^ A filter inlet for gases and aerosol (FIGAERO, Aerodyne Research Inc.) inlet system coupled with a high-resolution time-of-flight iodide chemical ionization mass spectrometer (HR-ToF-CIMS, Tofwerk Inc.) is used to detect both gas- and particle-phase molecular composition.^35^ Specifically, particles from chamber experiments are collected onto a PTFE filter (Pall Corp, Zefluor 25 mm, 2 μm pore-size; or Millipore Sigma, 25 mm, 5 μm pore-size) during the 20 min gas phase sampling. Molecular composition and volatility of particles are measured by thermally desorbing the collected particles using a set of programmed temperature ramp, soak, and cool steps, executed for 40 min.^32,36^

### 2.3 Estimation of Product Volatility

In each experiment, FIGAERO-CIMS is used to detect a suite of oxidized organic products in gas and particle phases. To investigate the volatility of the products formed, we estimate saturation mass concentrations (log_10_C*, μg/m^3^) of oxidized organic products using gas-particle partitioning, formula (i.e., SIMPOL), and thermogram methods from FIGAERO-CIMS data, as detailed in Stark et al.^37,38^ In the partitioning method, we calculate the log_10_C* of each product detected in both gas and particle phases using the following equation^39,40^
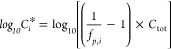
1where partitioning coefficient ; particle_*i*_ is the gas equivalent of the integrated particle signal of species *i* and gas_*i*_ is the average of gas-phase signal of species *i* without vapor wall loss correction during the peak SOA mass concentration cycle; *C*_tot_ is the organic mass loading (obtained by multiplying SMPS volume concentration and SOA density, see Section 3.1). It is noted that this method can only calculate the log_10_C* of products that exist in both phases to avoid *f*_*p*,*i*_ being zero or one, which will both lead to invalid log_10_C*. In the formula method, we apply the established volatility estimation method SIMPOL, which estimates log_10_C* values based on known contributions of different functional groups to volatility of products (i.e., group contribution approach).^38^ It is noted that while we use the proposed chemical structures of compounds measured in this study (Section 4.1), the estimated log_10_C* values might be affected by isomers with different structures. After log_10_C* of each product is obtained from the partitioning or the formula method, we calculate the average log_10_C* of all products that are present in both gas and particle phases weighted by their respective signal intensities to represent the overall volatility of products in each condition.^32,36^ In the thermogram method, we follow the approach detailed in prior studies.^32,36,37^ Briefly, a desorption temperature corresponding to the signal peak (*T*_max_) is calibrated using compounds with known vapor pressures. The FIGAERO-CIMS peak shape of the calibrated compounds is used to deconvolute the FIGAERO-CIMS signal of the SOA using nonlinear multipeak fitting and to obtain the volatility of the collected aerosol. It is noted that unlike the partitioning or the formula method, the log_10_C* values obtained from the thermogram method correspond to products that exist in the particle phase.

## 3 Results

### 3.1 SOA Yield under Different Oxidation Conditions

**Figure 1:**
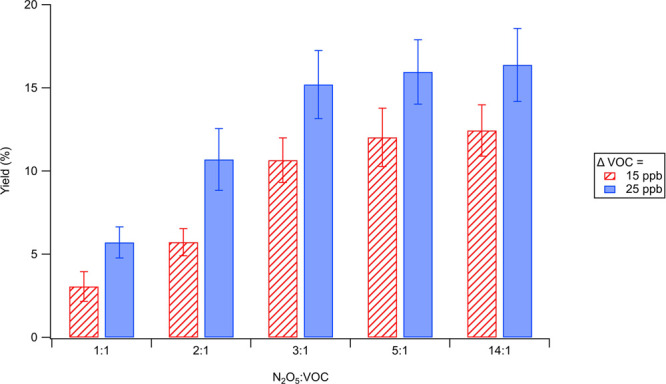
SOA mass yield for experiments with differing N_2_O_5_ to isoprene ratio. The error bars represented are propagated from uncertainties in Δ*M*_o_ and ΔVOC.

The effects of different oxidation conditions on SOA formation are examined by the variation in SOA yield, calculated as the ratio between the organic aerosol mass formed (Δ*M*_o_) and the mass of isoprene reacted (ΔVOC).^41^ For each experiment, we obtain Δ*M*_o_ by converting the aerosol volume concentration from SMPS to mass concentration using a SOA density of 1.42 g/cm^3^ as reported in a prior study.^26^Figure 1 and Table 1 show the Δ*M*_o_ and corresponding SOA yields (particle wall loss corrected) from both sets of experiments (15 and 25 ppb ΔVOC).^42^ The yields are not corrected for vapor wall loss. As vapor wall loss tends to decrease SOA yield because the semivolatile compounds might condense onto chamber walls instead of pre-existing particles to form SOA, the yields reported here are likely a lower bound.^43^ In the 25 ppb ΔVOC set (set A), the yield varies from 5.7% (Δ*M*_o_ = 4.2 μg/m^3^) to 16.3% (Δ*M*_o_ = 11.9 μg/m^3^). In the 15 ppb ΔVOC set (set B), the yield varies from 3.1% (Δ*M*_o_ = 1.2 μg/m^3^) to 12.4% (Δ*M*_o_ = 5.4 μg/m^3^). The SOA yields observed in both sets of experiments are similar to previous studies.^26,44,45^ However, the effect of changing N_2_O_5_/VOC ratio on the SOA yield has not been explored systematically before.

For both sets of experiments, the SOA yield is found to be dependent on the N_2_O_5_/VOC ratio. The NO_3_ concentration in the experiments is estimated with a 0-D model (framework for 0-D atmospheric modeling, F0AM), and the results are shown in Figure S1.^46^ Specifically, an increase of up to 300% in SOA yield is observed when N_2_O_5_/VOC increases from 1:1 to 3:1 in both experiment sets, as illustrated in Figure 1. Beyond 3:1, the SOA yield rises at a slower rate of only less than 2% when the N_2_O_5_/VOC ratio further increases.

### 3.2 Speciated Gas- and Particle-Phase Chemical Composition

**Figure 2:**
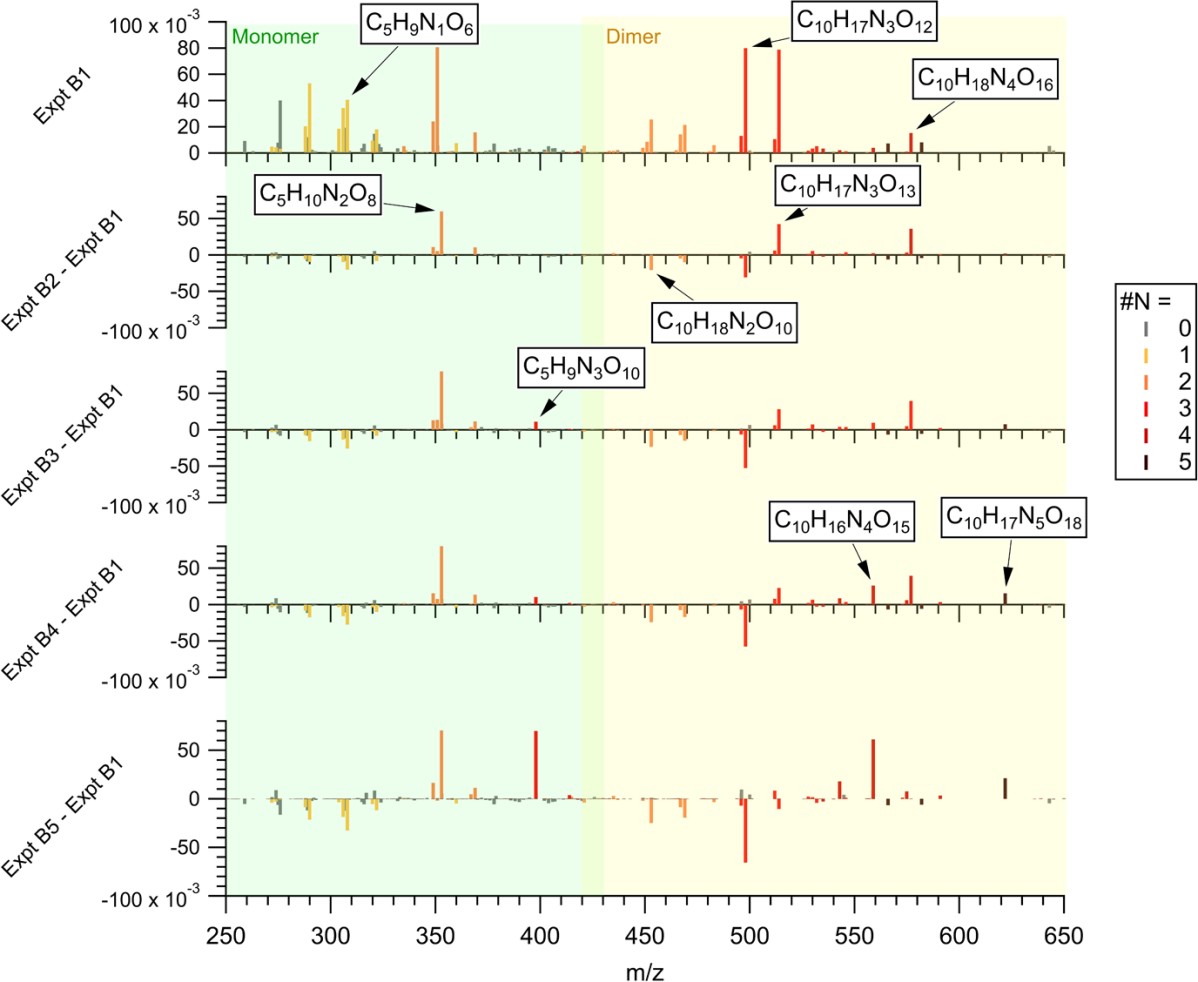
Typical particle-phase FIGAERO-CIMS mass spectrum (experiment B1) and comparison with other experiments (B2–B5). The vertical *y*-axes represent the signal of each compound normalized to the total organic compounds measured (arbitrary units). Monomer and dimer regions are shaded in green and yellow, respectively. Major species are labeled with the corresponding molecular formula.

**Figure 3:**
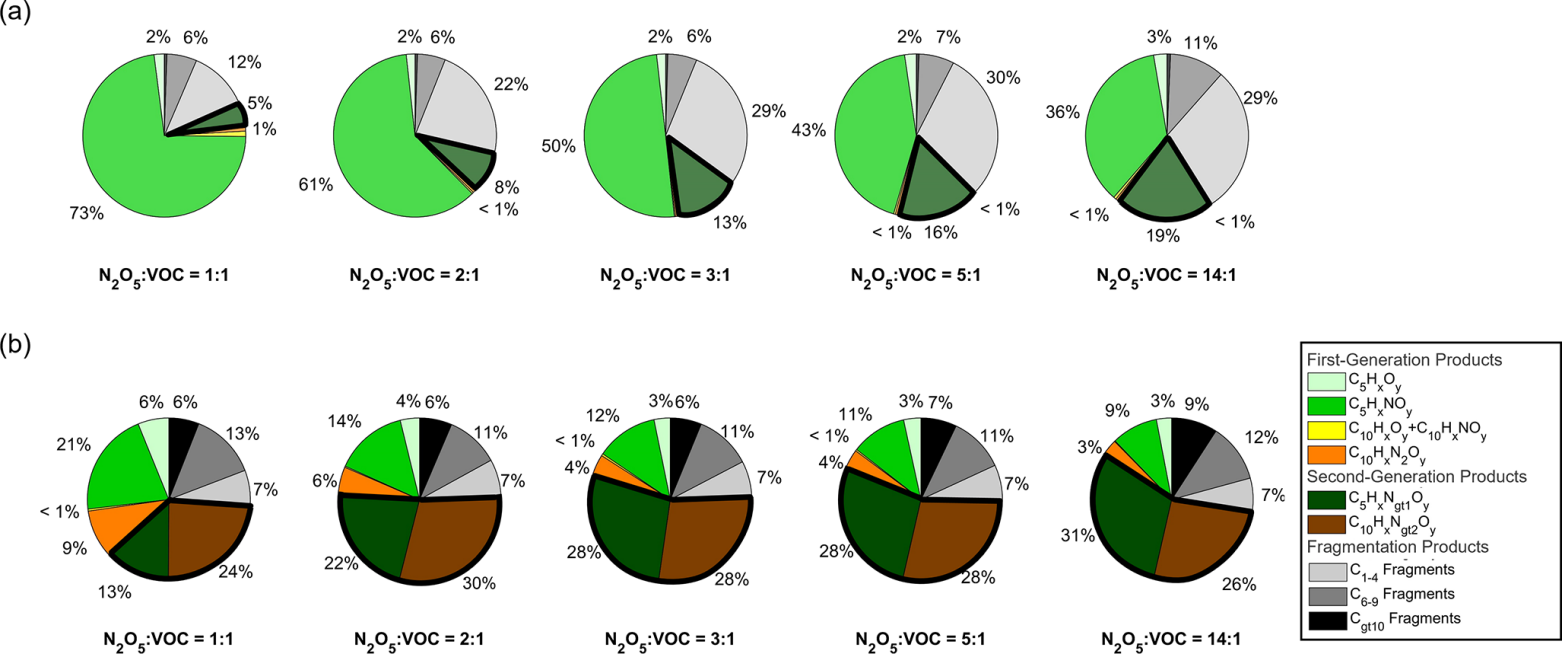
(a) Measured gas-phase product distributions classified into chemical families for experiments B1–B5. (b) Measured particle-phase product distributions classified into chemical families for experiments B1–B5. Both (a,b) are measured using FIGAERO-CIMS. The bold parts of the pie charts represent second-generation products.

In this study, FIGAERO-CIMS offers insights into the chemical composition of both gas- and particle-phase products, which allows us to link the gas-phase chemistry to SOA formation through gas-particle partitioning. The mass spectra of gas- and particle-phase products obtained from FIGAERO-CIMS at the peak SOA mass concentration cycle in each experiment are shown in Figure 2 (particle-phase mass spectra in set B), Figure S2 (particle-phase mass spectra in set A), and Figure S3 (gas-phase mass spectra in set A and set B). Both monomers (C_5_) and dimers (C_10_) are observed in both gas and particle phases under all experimental conditions, with a wide range of nitrogen number in their formulae: monomers can contain up to three nitrogen atoms and dimers can contain up to five nitrogen atoms. To compare the products in the gas phase and particle phase as well as across experiments with different N_2_O_5_/VOC ratios, we classify the products into monomer families of C_5_H_*x*_O_*y*_, C_5_H_*x*_NO_*y*_, and C_5_H_*x*_N_gt1_O_*y*_ (monomers with nitrogen atoms greater than one) and dimer families of C_10_H_*x*_O_*y*_, C_10_H_*x*_N_2_O_*y*_, and C_10_H_*x*_N_gt2_O_*y*_ (dimers with nitrogen atoms greater than two) as in Wu et al.^29^ The bulk product distributions from both phases are illustrated in Figure 3 for set B and Figure S4 for set A. As the distributions in both sets are similar, we simply focus on discussing the results from set B. It is noted that the particle-phase products measured by FIGAERO-CIMS might be subjective to thermal decomposition effect of the instrument: for example, the monomers measured from the instrument might be either the parent molecules (i.e., monomers) or decomposition fragments from dimers, as shown in Figure S5.^37^

Our gas-phase results are comparable to the distributions reported in Wu et al.^29^ Specifically, 1N-monomers (C_5_H_*x*_NO_*y*_ family) are the major gas-phase products, especially under low N_2_O_5_/VOC conditions. Their contribution is as high as 73% for N_2_O_5_/VOC of 1:1 and decrease to 36% for N_2_O_5_/VOC of 14:1. On the other hand, the contribution from higher nitrogen-containing monomers (C_5_H_*x*_N_gt1_O_*y*_ family) increases from 5% to 19% as more NO_3_ is available in the experiments. Meanwhile, the contribution of gas-phase dimers is minimal in all experiments, comprising less than 1% of the total signal.

The product distribution in the particle phase is very different from that in the gas phase. Compounds from both C_5_H_*x*_N_gt1_O_*y*_ family and C_10_H_*x*_N_gt2_O_*y*_ family are the major products in the particle phase. The fraction of products from these two families becomes higher while the fraction of C_5_H_*x*_NO_*y*_ family (1N-monomers) and C_10_H_*x*_N_2_O_*y*_ family (2N-dimers) decreases as N_2_O_5_/VOC increases. Specifically, the abundance of the C_5_H_*x*_N_gt1_O_*y*_ family rises from 13% to more than 30% as N_2_O_5_/VOC increases from 1:1 to 14:1 (Figures 3 and S4). Among specific products in the C_5_H_*x*_N_gt1_O_*y*_ family and C_10_H_*x*_N_gt2_O_*y*_ family, we also observe a shift toward products with even higher nitrogen atoms when N_2_O_5_/VOC ratio rises. For example, in Figure 2, monomers with three nitrogen atoms (e.g., C_5_H_9_N_3_O_10_) and dimers with four nitrogen atoms (e.g., C_10_H_16_N_4_O_15_) are present in higher fractions compared to other products with lower number of nitrogen atoms (e.g., C_5_H_10_N_2_O_8_ and C_10_H_17_N_3_O_12_) in the same C_5_H_*x*_N_gt1_O_*y*_ family and C_10_H_*x*_N_gt2_O_*y*_ family. To obtain an average representation of the aerosol composition measured with FIGAERO-CIMS, we also calculate the effective chemical formulae of particle-phase products formed under each condition, defined as the signal-weighted average of carbon, hydrogen, nitrogen, and oxygen numbers of all products (C, H, O, N > 0) at peak SOA mass concentrations (Table 1). Across all conditions, the effective formulae are bounded by the value of C_7–8_H_12–13_N_1–3_O_8–11_.

### 3.3 Product Volatility

The volatility of products that are present in both the gas phase and particle phase, calculated based on the partitioning method described in Section 2.3, is investigated and compared against related literature values for isoprene + NO_3_ oxidation products. Table 1 provides a summary of the product volatility (log_10_C*) in all experiments. Again, the average log_10_C* is calculated as the volatility of individual compounds that are present in both the gas and particle phases weighted by their respective signal intensities. The values of log_10_C* are similar (2.4–3.0) across all conditions for both sets of experiments, potentially due to the small change in effective formulae, as described in Section 3.2. These log_10_C* values are consistent with previously reported values for products formed from isoprene + NO_3_ chemistry using the same volatility estimation method.^44,45^

**Figure 4:**
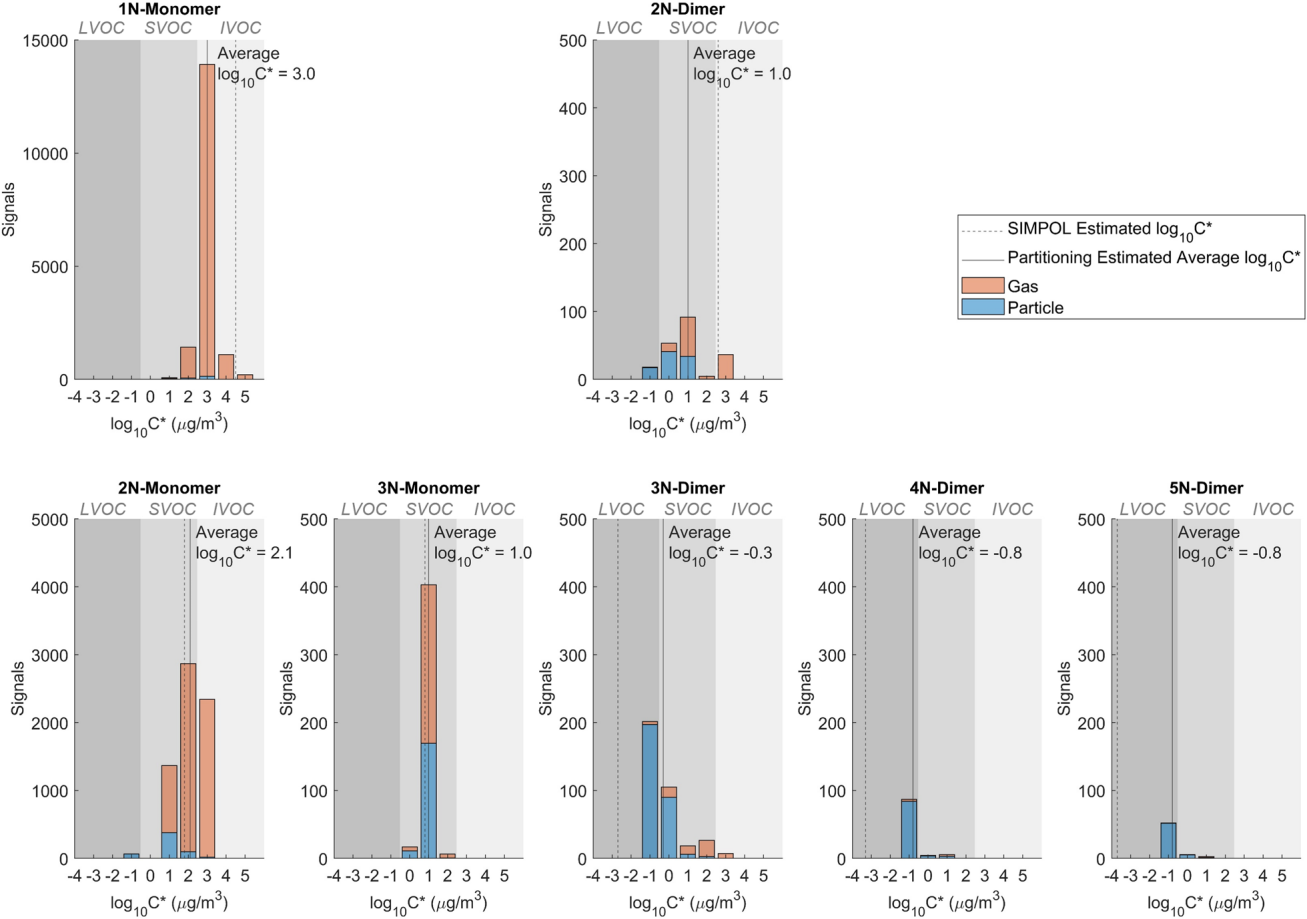
Product volatility distributions (experiment B4) calculated using the partitioning method and formula (i.e., SIMPOL) method. Each panel represents a different chemical family. The first row represents first-generation products. The second row represents second-generation products. Each shade of gray (from light to dark) represents IVOC, SVOC, and LVOC, respectively.

Apart from bulk volatility, we also investigate the volatility of specific products for each individual chemical family, as described in Section 3.2. We illustrate the results of the partitioning method and the formula method (Figure 4), as well as the thermogram method (Figure S6) by using data obtained in experiment B4. All three volatility estimation methods display the same trends between first- and second-generation products (i.e., first-generation products are more volatile than second-generation products). However, the thermogram method leads to unrealistically low volatility estimation results, in which the volatility of monomers is even lower than that of dimers (Figure S6). It is possible that some of the monomers are decomposition products as the –OO– bonds in dimer structures are susceptible to the thermal decomposition effect, resulting in monomers having higher than expected *T*_max_ values.^37^ On the other hand, the partitioning and formula methods offer similar trends and results in general, except for 3N-, 4N-, and 5N-dimers (Figure 4). Such discrepancy between the partitioning and the formula methods are consistent with literature, which might be a result of thermal decomposition, isomers, or instrument uncertainty.^37^ Therefore, while the thermogram method is more commonly used, we primarily focus on volatility estimation results from the partitioning method and the formula method in this work.^37^

## 4 Discussion

### 4.1 Multigeneration Chemistry and Product Distribution

**Scheme 1:**
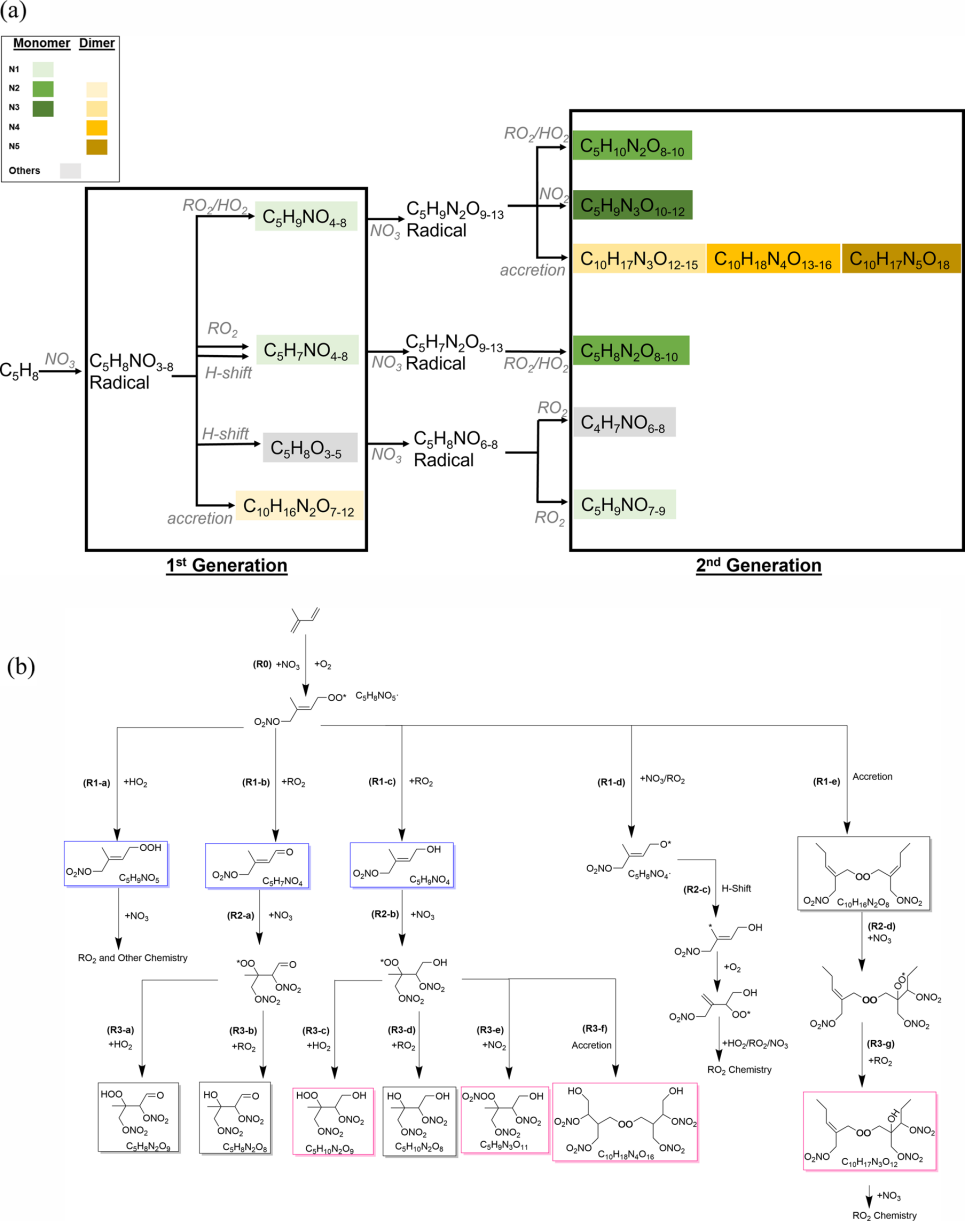
(a) Simplified RO_2_ Chemical Mechanisms and SOA Formation Pathways of Isoprene + NO_3_ Oxidation; The Colors of the Color Blocks Represent Nitrogen and Carbon Numbers of the Products as Shown in the Legend; (b) Proposed Formation Mechanisms of First- and Second-Generation Oxidation Products The boxed species are closed shell products, and the unboxed species are radicals. The box colors, blue, pink, and black, correspond to species detected only in the gas phase, only in the particle phase, and in both phases, respectively.

Previous literature have proposed the isoprene + NO_3_ gas-phase oxidation chemical mechanism, and our measurements are consistent with their findings, as summarized in Scheme 1.^26,29,44,45,47−50^ Since NO_3_ attacks carbon atoms in double bonds, the two double bonds of isoprene allow for multigeneration oxidation to occur. When NO_3_ adds to either of the two double bonds of isoprene, a first-generation RO_2_ (C_5_H_8_NO_5_) is formed (Scheme 1, R0). This RO_2_ can form first-generation products 0N-hydroxycarbonyls, 1N-monomers (e.g., C5-hydroxynitrate), 2N-dimers (e.g., C10-dihydroxy-dinitrate), or alkoxy (RO) radicals as it reacts with different radicals [R1(a–e)]. The first-generation products still have one unreacted double bond available for further oxidation to form second-generation products. The addition of NO_3_ to first-generation products results in the formation of second-generation RO_2_. The analogous RO_2_ chemistry that leads to the formation of first-generation products can take place for second-generation RO_2_, yielding 2N-, 3N-monomers and 3N-, 4N-, and 5N-dimers as second-generation products shown in R2 and R3 in Scheme 1.^29,50^ H-shift reactions can result in the formation of products with the same carbon, hydrogen, and nitrogen atom numbers but different oxygen atom numbers, as exemplified in Scheme S1. Overall, as both gas- and particle-phase products are measured in this study, we color the detected products in (1) the gas phase only, (2) the particle phase only, and (3) both the gas and particle phases in Scheme 1. The first-generation monomers tend to stay in the gas phase and do not partition into the particle phase, whereas other products either exist in both phases (i.e., first-generation dimers and second-generation monomers) or only in the particle phase (i.e., second-generation monomers and dimers). The volatility of the products that controls to what extent they will partition into the particle phase will be discussed in detail in Section 4.2.

There are many nitrogen-containing organic products formed in the reaction. Most of those products are organic nitrates (RONO_2_), such as 1N- and 2N-monomers, 2N-, 3N-, and 4N-dimers, and they can partition into the particle phase to form particulate organic nitrates (pON), as illustrated in Figures 2 and 3.^4,26,29,50^ To quantify the amount of pON formed, we use measurements obtained from both AMS and FIGAERO-CIMS. In AMS measurements, we measure organic nitrates based on the NO_2_^+^/NO^+^ ratio (NO_*x*_ ratio) because inorganic and organic nitrates fragment into NO_2_^+^ and NO^+^ in AMS differently (Figure S7).^4,51^ We report the NO_*x*_ ratio of organic nitrates, ammonium nitrate, and the corresponding ratio-of-ratios in Table S1. Using the data and method illustrated in Section S2, we determine the fraction of organic nitrates to total organic aerosol (pON/OA, in which pON includes the organics part and nitrate part of the organic nitrates and OA includes nitrated and non-nitrated organics of organic aerosol) to be over 70% across all conditions shown in Figure S8a, which is higher than field campaign values (e.g., 15–30%, from Takeuchi et al.) due to the presence of more non-nitrated organics in ambient mixtures.^4,31,52^ The trends of the total bulk pON measured by the AMS and the speciated pON measured by the FIGAERO-CIMS agree well, as shown in Figure S8b. If we assume all speciated pON have the same sensitivity, the sensitivity of pON calculated based on the two instruments (Table S2) across all conditions is found to be 5.18–8.91 Hz/ppt. Overall, our values are comparable with literature for isoprene organic nitrates (i.e., 1N-monomer standard, isoprene hydroxynitrate, 8.5–34 Hz/ppt) but they are on the lower end of the reported values.^25,53^ Future studies can further evaluate how the pON sensitivity corresponds to multifunctional higher-generation products.

Apart from pON, nitrogen-containing organic products can also be peroxy nitrates (ROONO_2_) or peroxyacyl nitrates (PAN). We propose that 3N-monomers (except C_5_H_9_N_3_O_10_), which is 0.2–0.8% of total particle phase signal, can be ROONO_2_ or PAN formed from the reaction between RO_2_ and NO_2_. Although ROONO_2_ and PAN are not stable and can rapidly decompose back to RO_2_ and NO_2_, the abundance of NO_2_ in the experiments (from the decomposition of N_2_O_5_) could enhance the formation of these products. Since PAN has a longer thermal decomposition lifetime (i.e., ∼1 h) than peroxy nitrate (i.e., ∼1 s), we propose that PAN is more likely to represent the structure of 3N monomers (except C_5_H_9_N_3_O_10_).^54^ On the other hand, since C_5_H_9_N_3_O_10_ contains too few oxygen atoms to be ROONO_2_ or PAN, it could be formed from the direct addition of N_2_O_5_ to the double bond of an 1N-monomer, as proposed by Zhao et al. and shown in Scheme S2.^50^ The same reaction pathway of N_2_O_5_ addition can also explain the formation of 5N-dimer (C_10_H_17_N_5_O_18_) observed in this work. Further studies are required to verify this mechanism.

**Figure 5:**
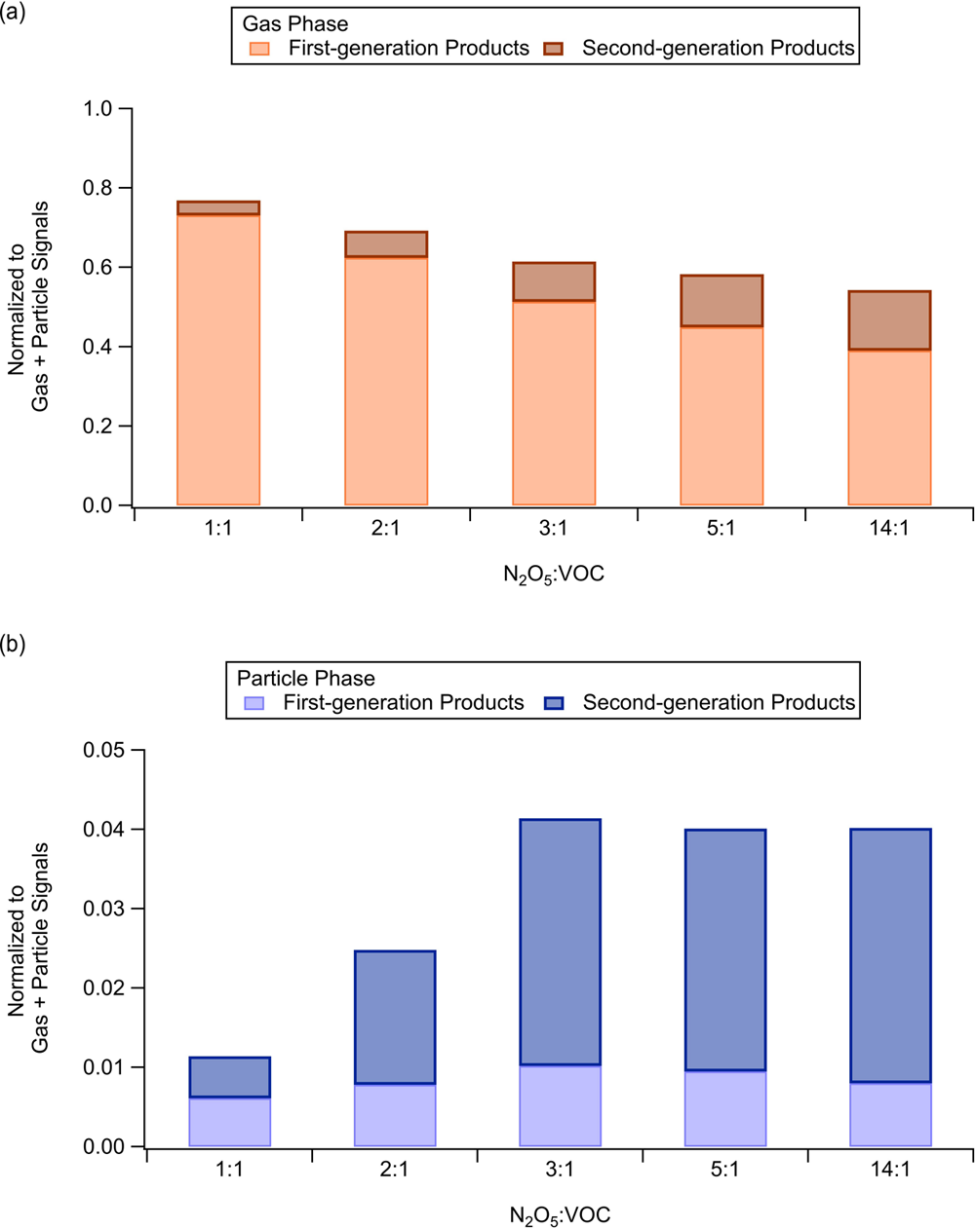
Distribution of bulk first- vs second-generation products in gas and particle phases for experiments B1–B5. (a) Signals of first- and second-generation products in the gas phase normalized by the sum of all products from both gas and particle phases. (b) Signals of first- and second-generation products in the particle phase normalized by the sum of all products from both gas and particle phases.

The variation of N_2_O_5_/VOC ratio leads to systematic shifts in the distribution of first- vs second-generation products in both gas and particle phases (Figures 2 and 3 and S3–S5). As the availability of NO_3_ increases, more first-generation products are reacted to form second-generation products. Time series of signature products (i.e., most abundant products) in each chemical family (Figure S9) confirm the decay of first-generation products and the continued formation of second-generation products. Moreover, the product distribution within the same generation can also be affected by NO_3_ availability in the system. We observe an increasing production of 3N-monomers and 5N-dimers (RO_2_ + NO_2_ pathway: R3-e, R3-g) and 4N-dimers (RO_2_ + RO_2_ pathway: R3-f) compared to other second-generation products (i.e., 2N-monomers and 3N-dimers), as illustrated in Figure 2, likely due to the higher NO_2_ and RO_2_ concentrations when the NO_3_ levels are higher. It is noted that since our FIGAERO-CIMS is an iodide-adduct CIMS, the sensitivity can vary drastically with structure of the compound, e.g., the addition of a single nitrate group tends to increase the sensitivity of the analyte.^55^ No study has investigated how the addition of more nitrate groups will impact instrument sensitivity. It is possible that first-generation and second-generation products reported in Figures 2, 3, and 5 might have different sensitivity in our measurements.

Though higher levels of NO_3_ promote further reactions of first-generation products, the conversion from first- to second-generation products is possible regardless of the N_2_O_5_/VOC ratio. It is interesting to observe that when the N_2_O_5_/VOC ratio is 1:1, a combination of first-generation and second-generation products is also observed, with the latter contributing to more than 30% of the products in the particle phase. In theory, when N_2_O_5_/VOC is 1:1, the formation of second-generation products should be minimal because there is not enough NO_3_ left to oxidize the first-generation products after they are consumed by isoprene. The presence of second-generation products in these experiments can arise from relatively fast reactions of first-generation products compared to isoprene oxidation. To the best of our knowledge, although the isoprene + NO_3_ reaction rate constant (*k*_VOC_) is known to be 6.5 × 10^–13^ cm^3^ molecules^–1^ s^–1^ at 298 K, that of the first-generation oxidation reaction (*k*_first_) in this system varies within a range from lower than 3 × 10^–15^ to 7 × 10^–14^ cm^3^ molecules^–1^ s^–1^.^45,56,57^ Thus, we conduct a simple sensitivity analysis using a 0-D model to constrain *k*_first_ with respect to *k*_VOC_ (Section S1).^46,54^ We find that when *k*_first_ is on the same order of magnitude as *k*_VOC_, (i.e., *k*_first_ = *k*_VOC_, Figure S10b) and higher than *k*_VOC_ (i.e., *k*_first_ = 100 × *k*_VOC_, Figure S10c), the modeled isoprene decay matches the measurements the best (Figure S10). According to the structure–activity relationship method proposed by Kerdouci et al., we estimate *k*_first_ of 1N-monomers and 2N-dimers to be on the order of 10^–11^–10^–12^ and 10^–12^–10^–13^ cm^3^ molecules^–1^ s^–1^, respectively (i.e., higher than or similar to reported k_VOC_).^58^ With this, the second-generation products can be formed even in limited-NO_3_ conditions owing to the fast oxidation of first-generation products which can compete with isoprene for the NO_3_ radical. Nevertheless, future studies shall further constrain the reaction rate constants of the large suite of first-generation products formed from isoprene + NO_3_ oxidation systematically.

### 4.2 Enhancement in SOA Yield as a Result of Multigeneration Chemistry and RO_2_ Fate

The variation in SOA yield as a function of N_2_O_5_/VOC ratio, as shown in Figure 1, can be a result of multigeneration chemistry. Compared to first-generation products, higher-generation ones are more likely to partition into the particle phase and contribute to SOA formation because they are more functionalized and less volatile. Figure 4 shows that second-generation products are generally 1–2 orders of magnitude lower in volatility than first-generation products with the partitioning method and up to 5 orders of magnitude lower with the formula method. The volatility distribution of first-generation products spans between intermediate-volatile organic products (IVOC, 2.5 < log_10_C* < 6.5) for 1N-monomers and semivolatile organic products (SVOC, −0.5 < log_10_C* < 2.5) for 2N-dimers. The volatility distribution of second-generation products spans between SVOC for 2N- and 3N-monomers and low-volatile organic products (LVOC, −4.5 < log_10_C* < −0.5) for 3N-, 4N-, and 5N-dimers.^59^ Therefore, most of the second-generation products only exist in the particle phase, as exhibited in Scheme 1 because they can readily partition into the particle phase. Previously, Wu et al. proposed that dimers are more likely to contribute to SOA formation compared to monomers based on differences in gas-phase measurements between nonseeded experiments and seeded experiments alone.^29^ However, with simultaneous gas and particle composition measurements in this work, we find that SVOC including second-generation products like 2N- and 3N-monomers can partition into the particle phase and form SOA. The differences in volatility between different generations of products allow us to explain why the SOA yield varies with the N_2_O_5_/VOC ratio. When we increase the N_2_O_5_/VOC ratio, more second-generation products with lower volatility are formed than first-generation ones, which then partition into the particle phase to form SOA, as summarized in Figures 5 and S11 and thus, SOA yield is enhanced when multigeneration reactions are promoted with higher N_2_O_5_/VOC ratio (Figure 1). A previous study suggested that modeling efforts should adapt the volatility of first-generation product 1N-monomer to represent the bulk volatility of SOA from NO_3_ oxidation of isoprene.^44^ However, we find that the volatility of second-generation 2N-monomers (average log_10_C* = 2.1 in Figure 4) is the closest to the bulk volatility (Table 1). Therefore, we suggest that future simulations should consider the variation in the product volatility when oxidation conditions are different. For example, the volatility of first-generation products might better represent bulk product volatility in isoprene-rich environments, whereas the volatility reported in this study could be more representative for NO_*x*_-rich environments.

Other than multigeneration chemistry, SOA yield can be affected by RO_2_ fate at the same time.^26^ We find that the extent of multigeneration reactions does not promote SOA yield infinitely. Once the NO_3_ level is higher than the amount needed to facilitate full multigeneration chemistry from reaction R0 to R3 (Scheme 1), the amount of SOA formed does not increase further and SOA composition also stays the same, as shown in Figures 1 and 3, respectively. For both sets of experiments, the SOA yields reach a maximum when N_2_O_5_/VOC = 3:1. If NO_3_ only reacts with the two double bonds of isoprene, the stoichiometric ratio of N_2_O_5_/VOC for complete reaction and the highest SOA yield would be 2:1. However, as shown in Scheme 1, first-generation RO_2_ (and similarly, second-generation RO_2_) can also react with NO_3_. To gain more insights into this, we calculate the first-generation RO_2_ fate [R1(a–e) in Scheme 1] using a 0-D model with kinetic data from Vereecken et al.^48^ Note that the model does not quantify the formation of second-generation products or second-generation RO_2_ fate [R2(a–d) and R3(a–g) in Scheme 1] owing to the lack of these specific reaction rate constants in the literature. More details are provided in Section S3.

The model results show that the RO_2_ + NO_3_ reaction accounts for 40–70% of the first-generation RO_2_ fate and competes with first-generation products for NO_3_ (Figure S12). To gain more understanding of the overall RO_2_ fate from both generations without available R2–R3 kinetic data, we then expand the model with the following assumptions: (1) *k*_first_ and *k*_VOC_ are equal, both are 1 order of magnitude slower than *k*_RO_2_+RO_2_/NO_3_/NO_2__, and (2) *k*_RO_2_+RO_2_/NO_3_/NO_2__ of first-generation and second-generation RO_2_ are similar. These assumptions allow us to apply the results of first-generation RO_2_ and related reactions (R0–R1) to those of second-generation RO_2_ and related reactions (R2–R3). Based on the 0-D model outputs, one molecule of NO_3_ is consumed when it oxidizes one molecule of isoprene to form first-generation RO_2_ (R0 in Scheme 1), which will further consume 0.4–0.7 molecule of NO_3_ [R1(d)] to form RO depending on the N_2_O_5_/VOC ratio of the experiment. When one molecule of the first-generation product is formed from other RO_2_ pathways, it will consume an additional molecule of NO_3_ to form second-generation RO_2_ (R2). Then, similar to reaction R1(d), another 0.4–0.7 molecule of NO_3_ will react with the second-generation RO_2_. Summing up the number of NO_3_ molecules required to reach complete oxidation of one molecule of isoprene, we will obtain the N_2_O_5_/VOC ratio of around 3:1 (instead of 2:1) because additional NO_3_ molecules are required to complete the multigeneration reactions to achieve the highest SOA yield (Figure 1) and a consistent product composition profile beyond the N_2_O_5_/VOC ratio of 3:1 (Figure 3). Generally, the maxima in SOA yield might not always occur exactly at a N_2_O_5_/VOC ratio of 3:1 because the above calculation depends on RO_2_ fate in different oxidation conditions. For example, RO_2_ fate can be different under ambient conditions in which more RO_2_ might undergo RO_2_ + RO_2_ reactions than RO_2_ + NO_3_ in chamber experiments. In this case, when one molecule of isoprene is reacted, less than 0.4–0.7 molecule of NO_3_ is required to react with second-generation RO_2_ and less than three molecules of NO_3_ is required to complete the multigeneration reactions, as described above for this study. Therefore, SOA maxima under that condition might take place at a N_2_O_5_/VOC ratio lower than 3:1. Further information such as kinetic data of second-generation RO_2_ chemistry will be needed to improve the constraints of the RO_2_ fate and facilitate a better linkage of radical chemistry to the SOA yield under different oxidation regimes in ambient or other laboratory conditions.

**Figure 6:**
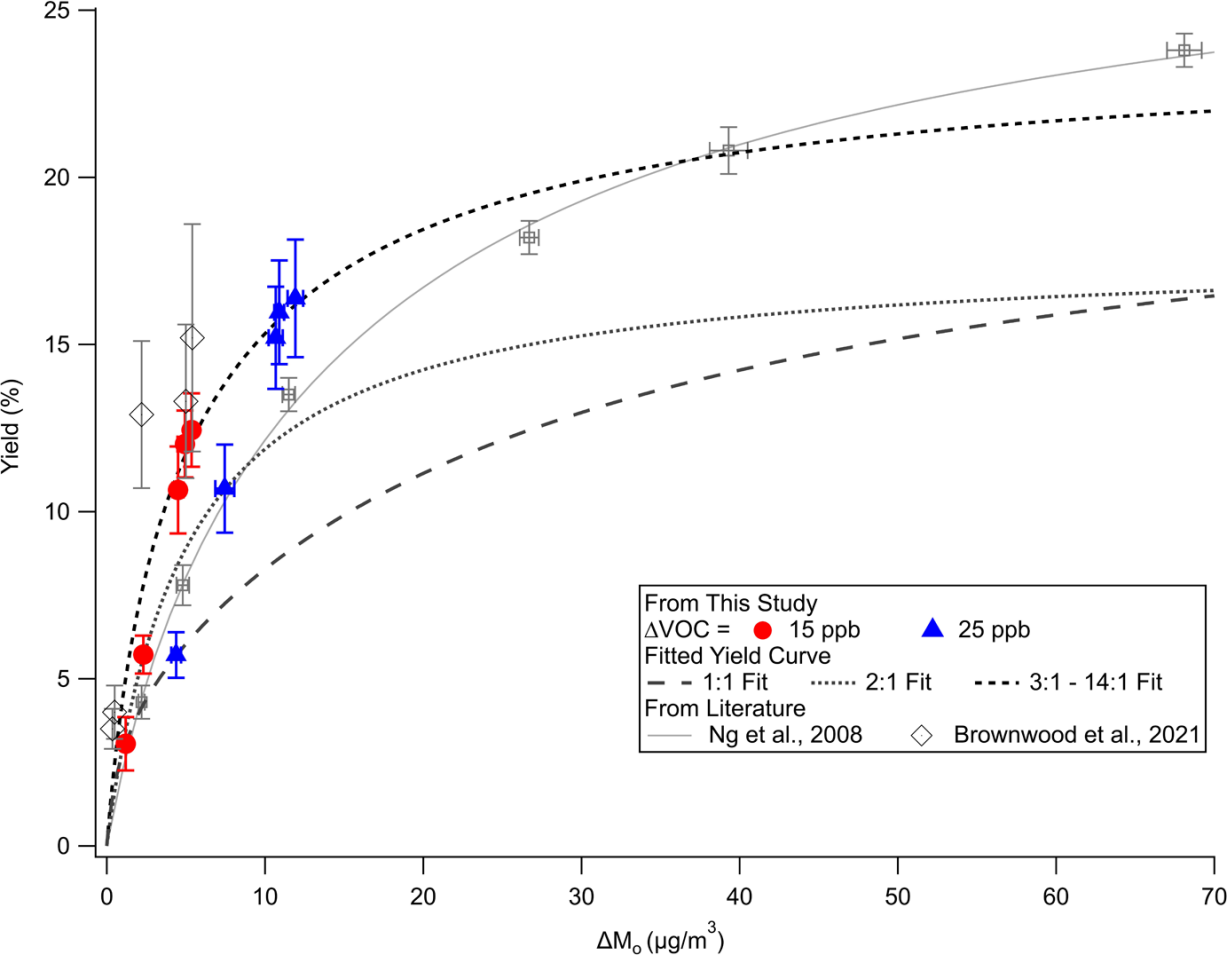
SOA mass yield as a function of organic mass loading for the isoprene + NO_3_ reaction from this work and literature.

Figure 6 shows the SOA yield data points and yield curves from this study as well as the yield data points and/or yield curve reported in studies by Ng et al.^26^ and Brownwood et al.^44^ which were calculated without vapor wall loss correction. Overall, the yield points from this study are comparable to results from the literature. Owing to the variation in yield and Δ*M*_o_ as a result of different oxidation conditions, we fit our data to three yield curves corresponding to different extents of multigeneration oxidation, as displayed in Figure 6 and Table S3: yield curves of (1) N_2_O_5_/VOC = 1:1, (2) N_2_O_5_/VOC = 2:1 (using data points in Table 1 and repeat experiments not shown), and (3) N_2_O_5_/VOC = 3:1–14:1 because the fraction of second-generation to first-generation products stays constant when N_2_O_5_/VOC ratio is equal to or higher than 3:1.^41^ The yield curve representing the highest degree of oxidation (i.e., 3:1–14:1) is higher than the other two yield curves as a result of SOA enhancement from multigeneration reactions. Ng et al. employed a high level of N_2_O_5_ (∼1 ppm) to enhance the extent of reaction and thus their yield curve is expected to be the most similar to our highest degree of oxidation experiments.^26^ For Δ*M*_o_ of about or less than 10 μg/m^3^, the yield points from our N_2_O_5_/VOC = 3:1–14:1 experiments are higher than those in Ng et al. at comparable Δ*M*_o_ values.^26^ As this study uses a much lower level of N_2_O_5_ and injects N_2_O_5_ at a much slower rate than the typical experiments of Ng et al. (N_2_O_5_/VOC = 10:1–50:1), it is likely that relatively more RO_2_ in this study undergo RO_2_ + RO_2_ reaction and thus form more ROOR, which can more readily partition to particle phase and enhance formation of SOA.^26^ Taken together, both RO_2_ fate and multigeneration chemistry can impact SOA yield, and one needs to take both factors into consideration when comparing SOA yield across different studies.

## 5 Implications

This is the first study to systematically investigate multigeneration chemistry in the isoprene + NO_3_ system using chemical composition measurements in both gas and particle phases. Our results show the effects of multigeneration chemistry on SOA formation: product composition, volatility, and SOA yield are all affected by different degrees of oxidation, depending on the availability of NO_3_ relative to isoprene. Kinetic modeling results show that first-generation products in this system are highly reactive and, with excess NO_3_, multigeneration chemistry can occur, forming increasing amounts of second-generation products. These second-generation products partition into the particle phase more readily than the first-generation ones because their volatility (log_10_C* = −0.8–2.1 using the partitioning method and log_10_C* = −3.7–1.8 using the formula method) is 1 to 5 orders of magnitude lower than the volatility of first-generation products (log_10_C* = 1.0–3.0 using the partitioning method and log_10_C* = 2.6–4.5 using the formula method). As a result, excess NO_3_ leads to increased SOA yields, from 5.7% (Δ*M*_o_ = 4.2 μg/m^3^) to 16.3% (Δ*M*_o_ = 11.9 μg/m^3^) when ΔVOC is 25 ppb and from 3.1% (Δ*M*_o_ = 1.2 μg/m^3^) to 12.4% (Δ*M*_o_ = 5.4 μg/m^3^) when ΔVOC is 15 ppb.

While multigeneration chemistry is not typically reported in low oxidant environments in field studies (when N_2_O_5_/VOC = ∼1:100 according to the reported N_2_O_5_ and isoprene concentrations in literature), it can still take place when first-generation products are transported to other areas with higher abundance of NO_3_ radical or are able to compete against precursor VOCs for NO_3_ radical owing to their fast oxidation rates.^60,61^ In this study, we show that higher-generation products can not only form quickly but also partition into the particle phase more easily than mononitrates due to their lower volatility. In previous ambient measurements based in the southeastern U.S., only mononitrates (e.g., C_5_H_9_NO_7_) have been identified in the particle phase as products of isoprene + NO_3_ or isoprene + OH + NO_*x*_ reaction.^24,25^ There is limited discussion on the formation of di- or trinitrates from multigeneration chemistry other than a Shanghai-based campaign identifying certain isoprene oxidation products at nighttime to be second-generation dinitrates from isoprene + NO_3_ reaction (e.g., C_5_H_8_N_2_O_8_).^26,62^ Future field measurements can use the tracer compounds reported in our study including C_5_H_8,10_N_2_O_8_, C_5_H_9_N_3_O_10_, and C_10_H_17_N_3_O_13_ to evaluate the prevalence of multigeneration chemistry from the isoprene + NO_3_ reaction in polluted atmosphere and/or downwind of isoprene-rich environments.^63^ Further, the estimated fast reaction rate constants of first-generation products with NO_3_ and the volatility of the corresponding second-generation products provide new fundamental constraints for more accurate simulations of SOA formation from multigeneration NO_3_ radical oxidation of isoprene in different ambient conditions.

Although this study focuses primarily on the effects of multigeneration chemistry, we also find a SOA yield enhancement resulting from the coupling effects of RO_2_ fate and multigeneration reactions. In this study, SOA formation is enhanced primarily due to multigeneration oxidation. However, comparison of SOA yields against the literature for experiments with similar Δ*M*_o_ values highlights the role of RO_2_ fate in SOA formation. This work does not address the effects of RO_2_ fate in a comprehensive way as the experiments are not designed specifically to explore varying RO_2_ fates. Future studies should investigate the effects of both multigeneration oxidation and RO_2_ fate under atmospherically relevant conditions. Under ambient conditions, RO_2_ + RO_2_ chemistry is often more pronounced at nighttime and thus might result in an even higher SOA yield compared to the SOA enhancement shown in this study from multigeneration oxidation alone.^64,65^ For example, an ambient study from Fry et al. suggests that NO_3_ radical oxidation of isoprene can form SOA with yield up to 27% ± 14%, which is comparable to the highest yield (16% ± 2%) reported in this study with the comparable Δ*M*_o_ = 10 μg/m^3^, considering the uncertainty in yields in both studies. Finally, since the coupled effects of multigeneration oxidation and RO_2_ fate can make direct comparisons of SOA yields between different laboratory and field studies in literature difficult, such comparisons should be approached with caution, considering that multiple factors can be at play in determining SOA formation.

## Supporting Information Available

Additional information and details regarding 0-D modeling of isoprene decay, quantification of organic nitrates with AMS, FIGAERO-CIMS gas and SOA mass spectra, gas- and particle-phase products classified into chemical families, thermograms of speciated compound groups, SOA volatility estimation results using thermogram method and gas-particle partitioning method, AMS mass spectra of organic aerosol, comparison of organic nitrates in this work with literature, time evolutions of first- and second-generation products, comparison of modeled isoprene decay under different reaction rates with measured isoprene decay, distribution of bulk first- and second-generation products in gas and particle phases, comparison of peroxy radical fates across all conditions, summary of *R*_AN_ and *R*_ON_, and summary of pON sensitivity (PDF)
