# Supplementary material for: Multigeneration Chemistry in Secondary Organic Aerosol Formation from Nitrate Radical Oxidation of Isoprene
Source: ACS Earth Space Chem. 2025 Jan 27;9(2):411–23. doi: 10.1021/acsearthspacechem.4c00417 (PMC11849032; doi:10.1021/acsearthspacechem.4c00417)
Supplement: Supplementary file 1 — sp4c00417_si_001.pdf [file sp4c00417_si_001.pdf]

Supporting Information for

Multi-generation Chemistry in Secondary Organic Aerosol Formation from Nitrate Radical Oxidation of Isoprene

Tianchang Xu[1], Masayuki Takeuchi[2,a], Jean C. Rivera-Rios[1,b], Nga L. Ng[1,2,3*]

[1]School of Chemical & Biomolecular Engineering, Georgia Institute of Technology, Atlanta, Georgia 30332, United States

[2]School of Civil & Environmental Engineering, Georgia Institute of Technology, Atlanta, Georgia 30332, United States

[3]School of Earth & Atmospheric Sciences, Georgia Institute of Technology, Atlanta, Georgia 30332, United States

[a]Now at: Department of Mechanical Engineering, University of Colorado Boulder, Boulder, Colorado, 80309, United States

[b]Now at: Department of Chemistry & Chemical Biology, Rutgers University, Piscataway, New Jersey, 08854, United States

Table of Contents

S1. Modeling the Decay of Isoprene S3
S2. Formation of Organic Nitrates S4
S3. Modeling RO_2 Fate S6

Figure S1 .... S7
Figure S2 .... S8
Figure S3 .... S9
Figure S4 .... S11
Figure S5 .... S12
Figure S6 .... S13
Figure S7 .... S14
Figure S8 .... S16
Figure S9 .... S17
Figure S10 .... S18
Figure S11 .... S19
Figure S12 .... S20

Table S1 .... S21
Table S2 .... S22
Table S3 .... S23

Scheme S1 .... S24
Scheme S2 .... S25

S1. Modeling the Decay of Isoprene

The Framework for 0-D Atmospheric Modeling (F0AM) is conducted using experimental conditions in Table 1 to simulate the decay of isoprene.[1] We use the kinetic rate of isoprene-NO_3 reaction (k_{VOC} = 6.5×10^{-13} cm^3 $molecules^{-1}$ s^{-1} at 295K) and estimate that of reactions between first-generation products and NO_3 based on MCM v.3.3.1.[2] Although MCM offers a comprehensive set of kinetic rates, an issue is that the MCM reaction rate of RO_2+RO_2 is slower than compared to literature reported value.[3] Therefore, we increase this reaction rate from 1.3×10^{-12} (MCM value) to 5×10^{-12} cm^3 $molecules^{-1}$ s^{-1} which is the literature reported value.[3, 4] After close shell compounds are formed from RO_2 chemistry, MCM includes the formation of C5-dinitrate (NISOPNO3), C_5-nitrooxyhydroperoxide (NISOPOOH), and C_5-nitrooxycarbonyl (NC_4CHO) from the reaction of isoprene-derived RO_2 ($NISOPO_2$) as first-generation products. Among these three products, only the kinetic rate of NC_4CHO-NO_3 is reported in MCM (k_{1st} =1.2×10^{-14} in cm^3 $molecules^{-1}$ s^{-1} at 298K). The value of k_{VOC} has been validated by multiple prior studies and thus we only alter the value of k_{1st}.[3-5] We manually adjust the second-generational reaction rate k_{1st} to be 100 times, same, or 0.01 time of k_{VOC}, respectively. The simulated isoprene decays from these three scenarios are shown in Figure S9. It is noted that the losses of N_2O_5 and NO_3 to the chamber are not accounted for in the model but a recent study has suggested that loss rates of N_2O_5 and NO_3 to the wall are negligible compared to loss to oxidation reaction.[6] Therefore, we expect these loss processes to have minor impacts on the model outputs.

S2. Formation of Organic Nitrates

In this study, we quantify particulate organic nitrates using HR-ToF-AMS measurement. As proposed in previous literature, inorganic and organic nitrates fragment into NO_2^+ and NO^+ differently in the AMS.[7, 8] We calculate the Ratio-of-Ratios (RoR) introduced in Fry et al. as [9]

$$RoR = \frac{\left(\frac{NO_2^+}{NO^+}\right)_{AN}}{\left(\frac{NO_2^+}{NO^+}\right)_{expt}} \quad \text{(S1)}$$

where $\left(\frac{NO_2^+}{NO^+}\right)_{expt}$ is obtained from each experiment at peak SOA mass concentration and $\left(\frac{NO_2^+}{NO^+}\right)_{AN}$ is obtained from atomizing ammonium nitrate solution into the AMS during AMS ionization efficiency calibration (Table S1). As shown in Figure S7(c), RoR value in this study ranges from 1.26 to 1.80 where higher VOC:N_2O_5 conditions tend to display higher RoR values. Compared to literature, RoR in this study are different the reported values of organic nitrates formed from nitrate radical oxidation of isoprene: 2.24 (Rollins et al.) and 2.08 (Bruns et al.), and the average value of 2.75±0.70 reported in Day et al.[10-12]

The contrition of particulate organic nitrates (pON) to organic aerosol is calculated using the method described in Takeuchi and Ng as [13, 14]

$$\frac{pON}{OA} = \frac{NO_{2_{ON}}}{Org+NO_{3_{ON}}} * \frac{MW_{ON}}{MW_{NO\ ON}} \quad \text{(S2)}$$

where $NO_{2_{ON}}$ and Org represent the mass concentrations of nitrogen-containing moiety of $RONO_2$ and organics measured by HR-ToF-AMS, MW_{ON} represents the molecular weight of effective formulae of particulate organic nitrates per nitrate group functionality as

described in Section 3.2 and Table 1, $MW_{NO_{2_{ON}}}$ is the molecular weight of nitrate-containing moiety of 46 g/mol since organic nitrate fragments into NO_2 in HR-ToF-AMS.[14]

Since both HR-ToF-AMS and FIGAERO-CIMS measure pON, we can calculate the pON sensitivity in Hz/ppt by using the equation:

$$pON\ sensitivity\ corrected\ by\ AMS\ collection\ efficiency =$$

$$pON_{FIGAERO-CIM}\ /(\frac{pON}{OA} * \frac{OA}{MW_{ON}} * \frac{\frac{RT}{P}}{CE_{AMS}*1000}) \qquad \text{(S3)}$$

where $pON_{FIGAERO-CIM}$ is total signals of organic nitrates in the particle phase, OA and MW_{ON} are the same as Equation S2, $\frac{pON}{OA}$ is calculated from Equation S2, CE_{AMS} represents the collection efficiency of HR-ToF-AMS calculated in this study, R is ideal gas constant, T is temperature, and P is pressure. The denominator converts pON measured in AMS from μg/m^3 to ppt to stay consistent with unit in literature reported values.[15] The calculated results are shown in Table S2.

S3. Modeling RO_2 Fate

We use the same F0AM model described in Section S1 to model the RO_2 fate.[1] Instead of MCM, kinetic data from Vereecken et al., on isoprene+NO_3 oxidation are applied to the model.[5] We select this dataset over MCM, because MCM not only greatly underestimates the isoprene RO_2+RO_2 rate but also does not include the isomerization pathway of isoprene-derived RO_2.[2-4] Meanwhile, the model in this section does not consider any reaction further than R2 (i.e., oxidation of first-generation products) owing to a lack of specific kinetic data in literature.

In the model, isoprene is modeled as a point source injected at the beginning of experiment while N_2O_5 is modeled as a continuous emission source for different duration of injection times to represent the experiment conditions as accurately as possible. The RO_2 fate is calculated as the percentage of isoprene-derived RO_2 reacting with other RO_2, NO, NO_2, NO_3, HO_2, or going through unimolecular isomerization.

In both sets of experiments, RO_2+NO_3 is the dominant reaction pathway for first-generational RO_2 oxidation as illustrated in Figure S11. When NO_3 level increases from N_2O_5:VOC of 1:1 to 3:1, less RO_2 undergo RO_2+RO_2 reactions while more RO_2 undergo RO_2+NO_3 reactions instead. Beyond N_2O_5:VOC of 2:1 (Set A) or 3:1 (set B), RO_2 fate distribution stays relatively consistent. Comparing the experiments with different ΔVOC, RO_2 fate also changes: more RO_2 undergo RO_2 + RO_2 reactions even under the same N_2O_5:VOC ratio.

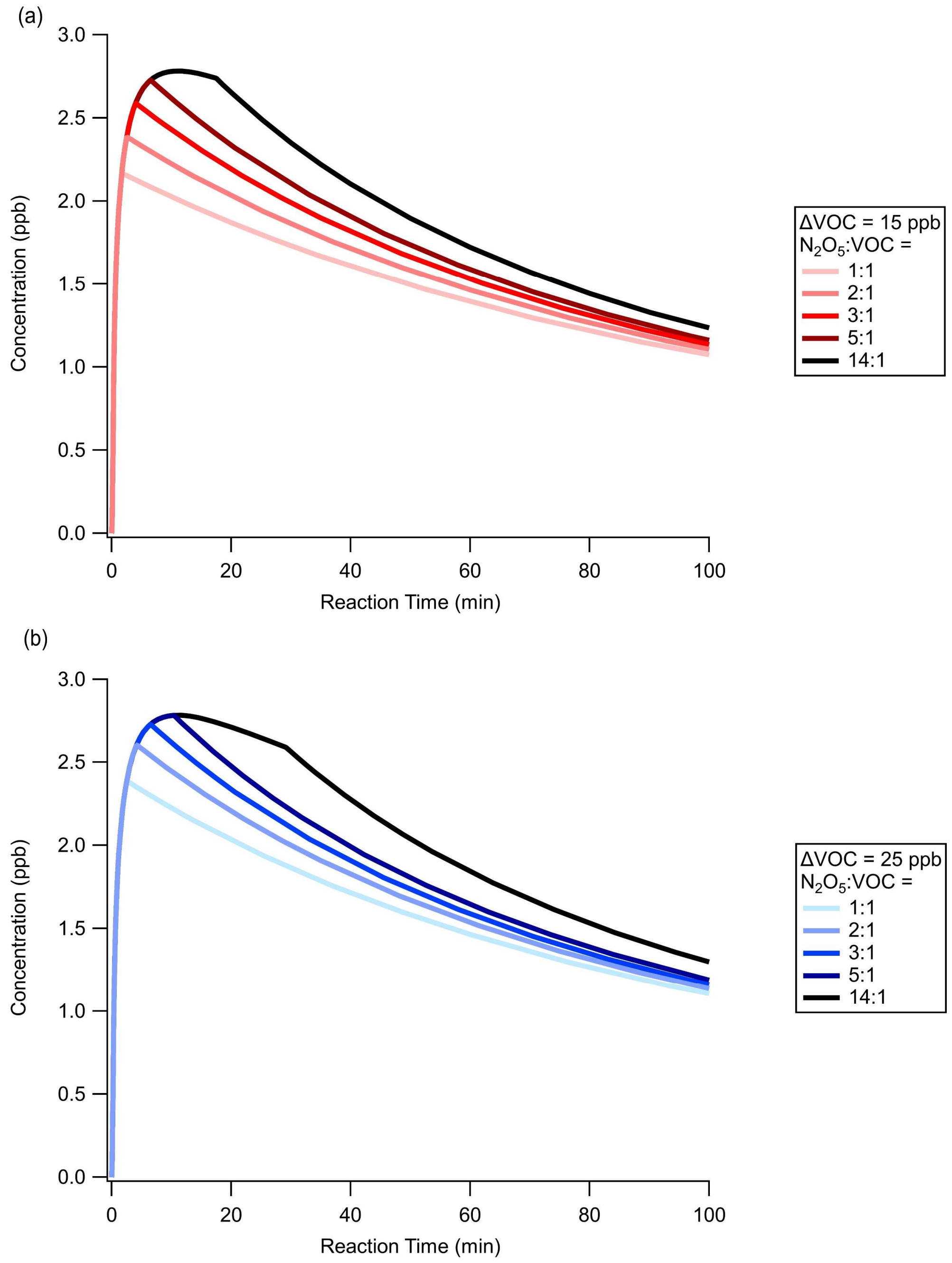

Figure S1. Modeled NO_3 concentration using 0-D model for (a) Set A and (b) Set B. The abrupt change in the time series corresponds to the time when N_2O_5 injection comes to completion in each condition. The plateau of N_2O_5 before its decay for high N_2O_5:VOC ratios represent the equilibrium between $NO_3 + NO_2 \rightleftharpoons N_2O_5$ due to the high concentration of NO_2 injected and NO_3 produced.

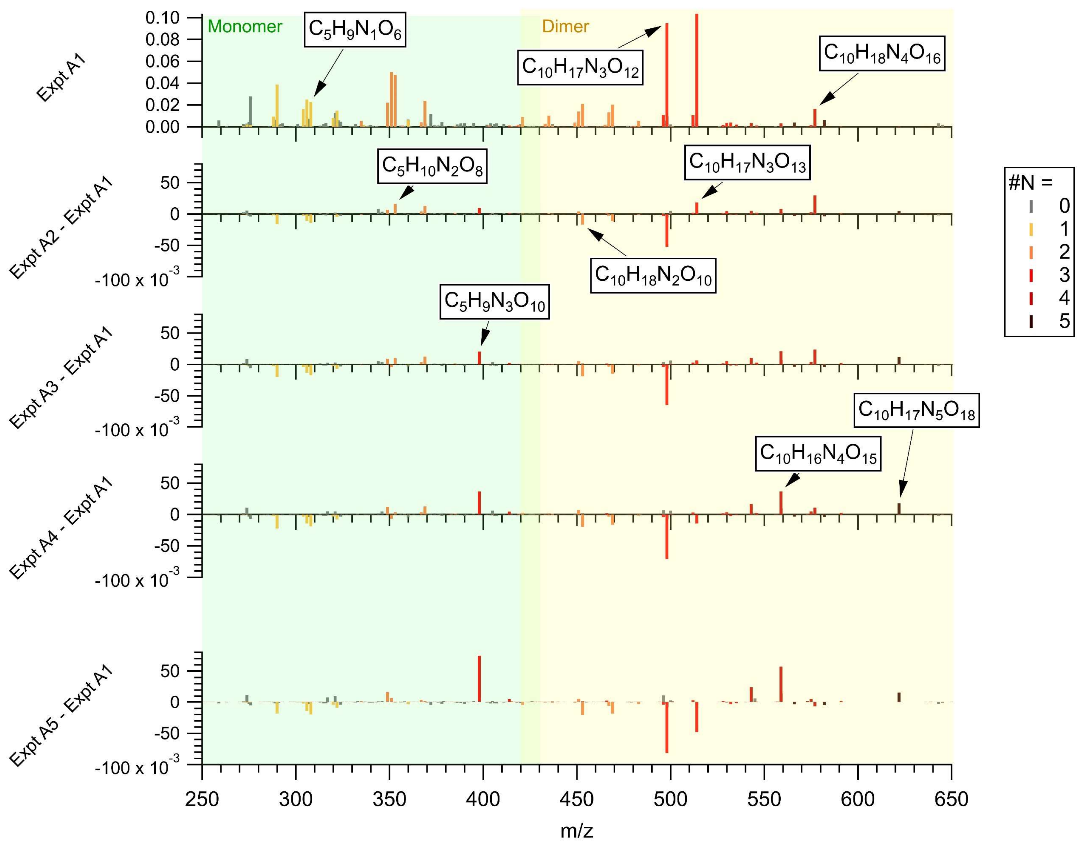

Figure S2. Comparison of FIGAERO-CIMS SOA mass spectra between experiment A1 and all other experiments in Set A (A2-A5). The y-axes represent the normalized signal intensities (arbitrary units). Monomer and dimer regions are shaded in green and yellow, respectively. Major species are labeled with the corresponding molecular formula.

(a)

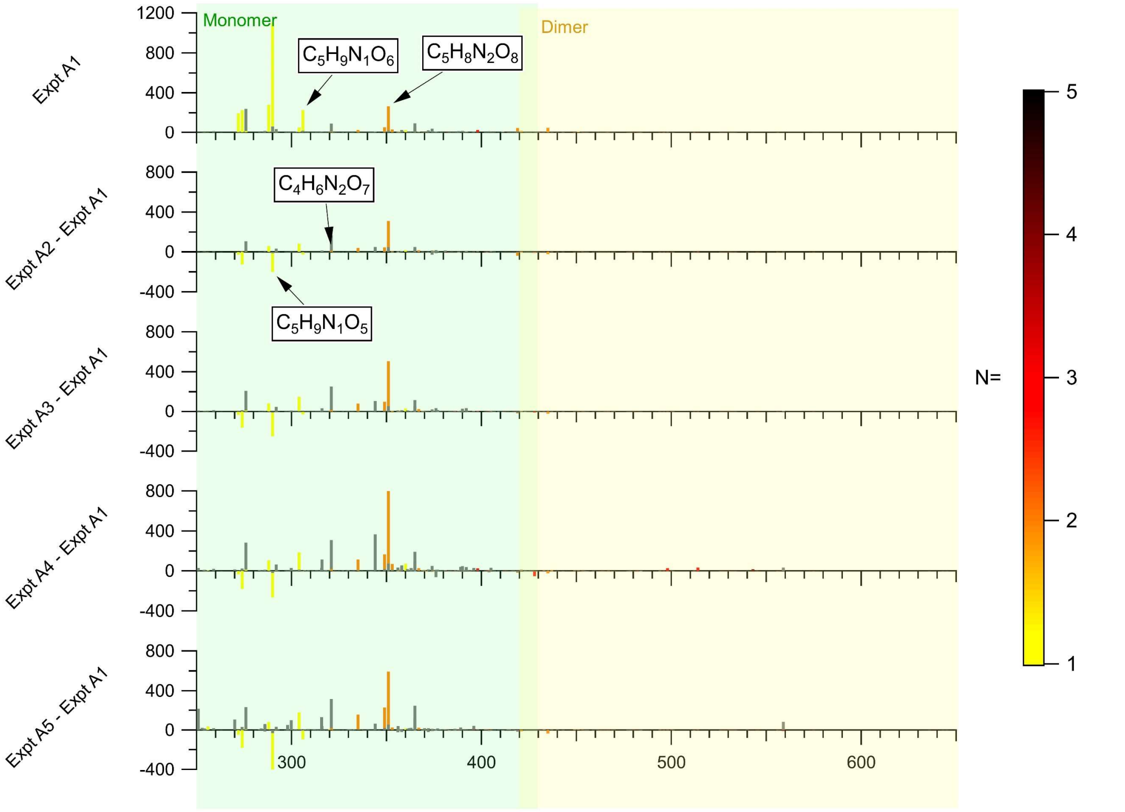

Monomer
Dimer
$C_5H_9N_1O_6$
$C_5H_8N_2O_8$
$C_4H_6N_2O_7$
$C_5H_9N_1O_5$
Expt A1
Expt A2 - Expt A1
Expt A3 - Expt A1
Expt A4 - Expt A1
Expt A5 - Expt A1
1200
800
400
0
-400
300
400
500
600
N=
1
2
3
4
5

(b)

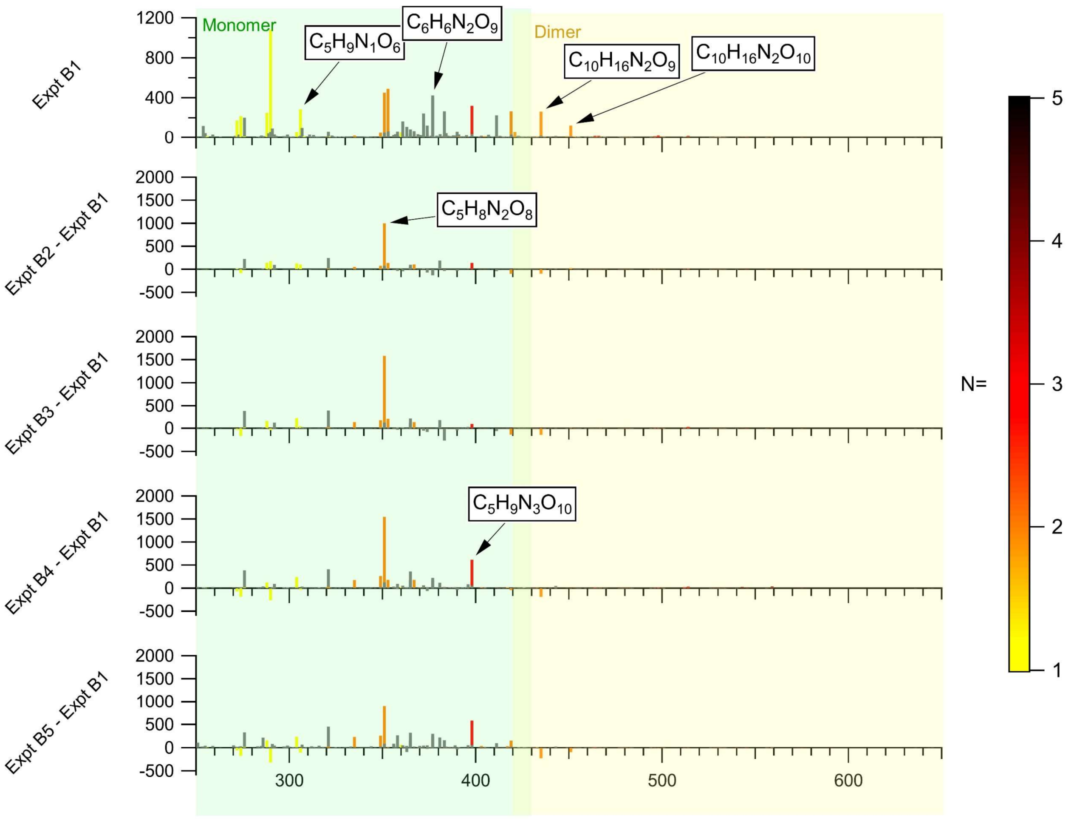

Figure S3. Comparison of FIGAERO-CIMS gas-phase mass spectra between experiment (a) A1 and all other experiments in Set A (A2-A5), (b) B1 and all other experiments in Set B (B2-B5).

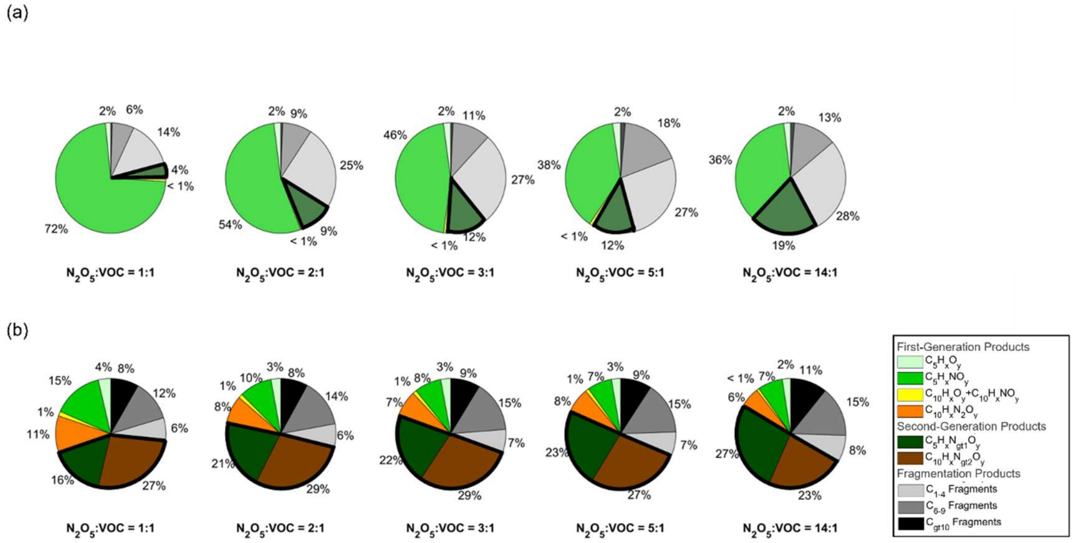

Figure S4. (a) Measured gas-phase product distributions classified into chemical families from experiments A1-A5 (b) Measured particle-phase product distributions classified into chemical families from experiments A1-A5. Both (a) and (b) are measured using FIGAERO-CIMS. The bolded parts of the pie charts represent second-generation products.

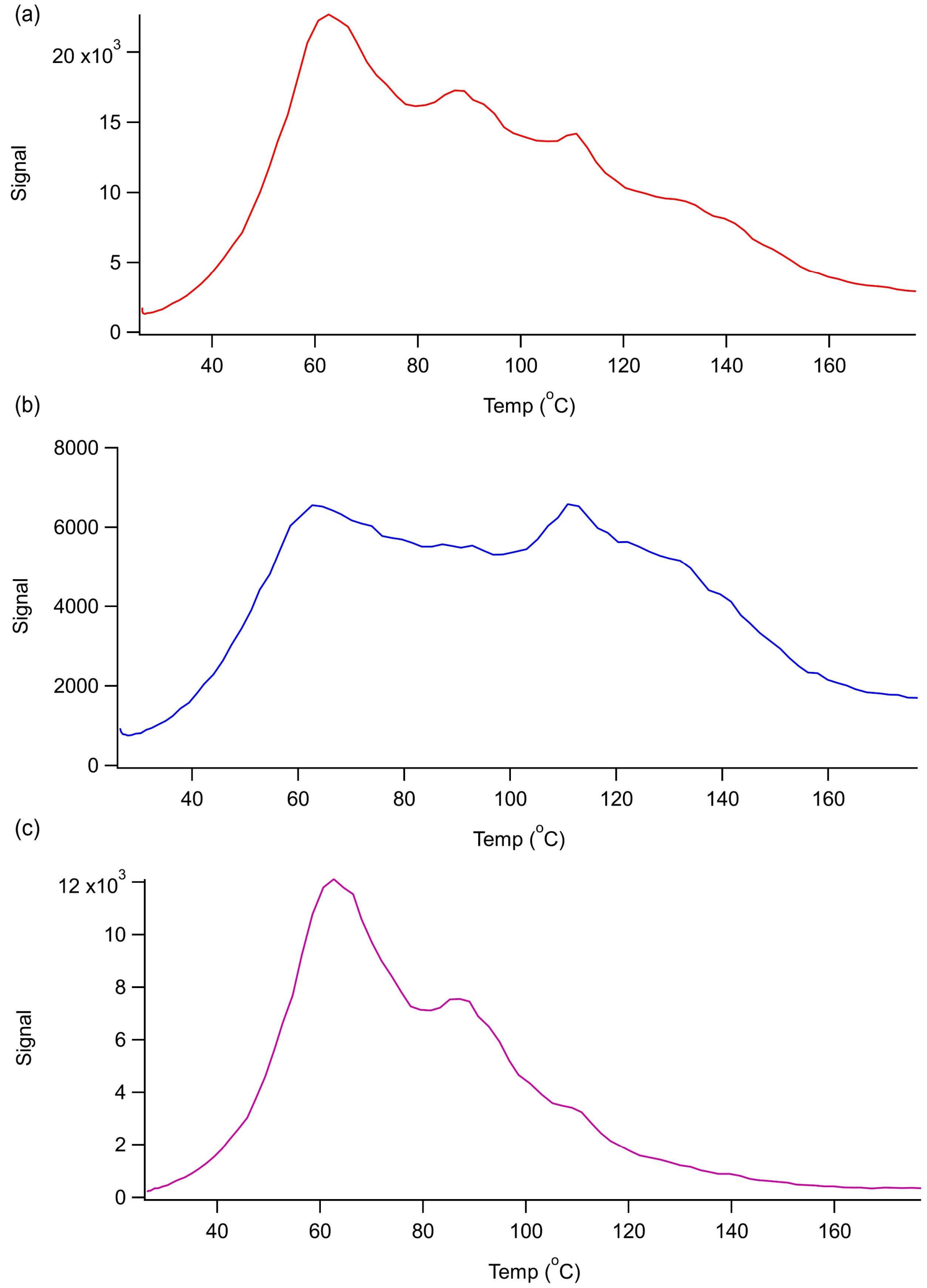

Figure S5. Raw thermograms for experiment A4 of (a) all organics, (b) monomers only, and (c) dimers only. Since dimers are formed from accretion reactions of two RO_2 radicals connected by a peroxide linkage (-OO-) in Scheme 1, which is a weak bond and can decompose easily at temperatures as low as 80 °C, monomers tend to display bi-modal peaks in the thermogram: the first peak likely corresponds with the thermal desorption profile of the monomer parent ion and the second peak likely corresponds with the thermal decomposition profile when dimers decompose into monomers. [16]

(a)

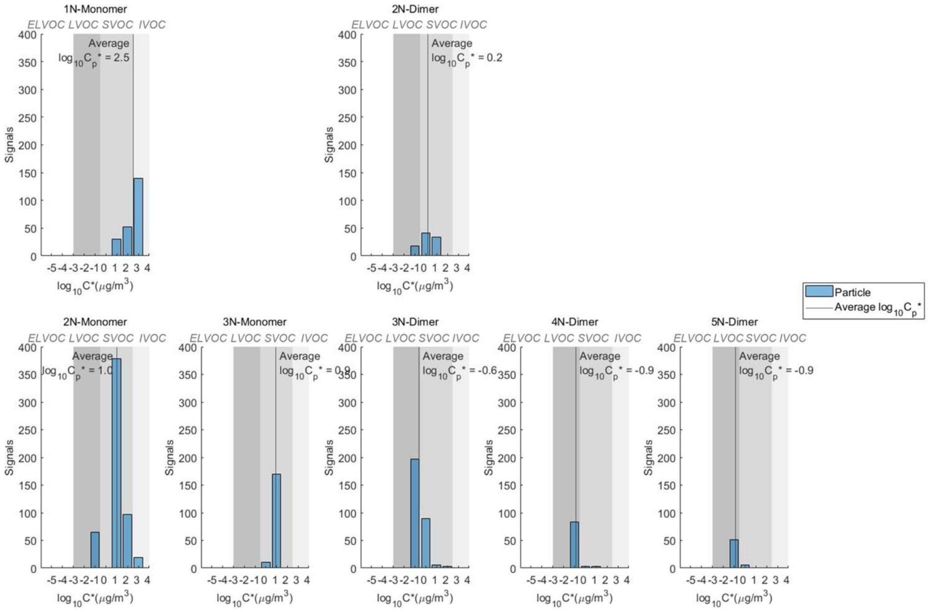

(b)

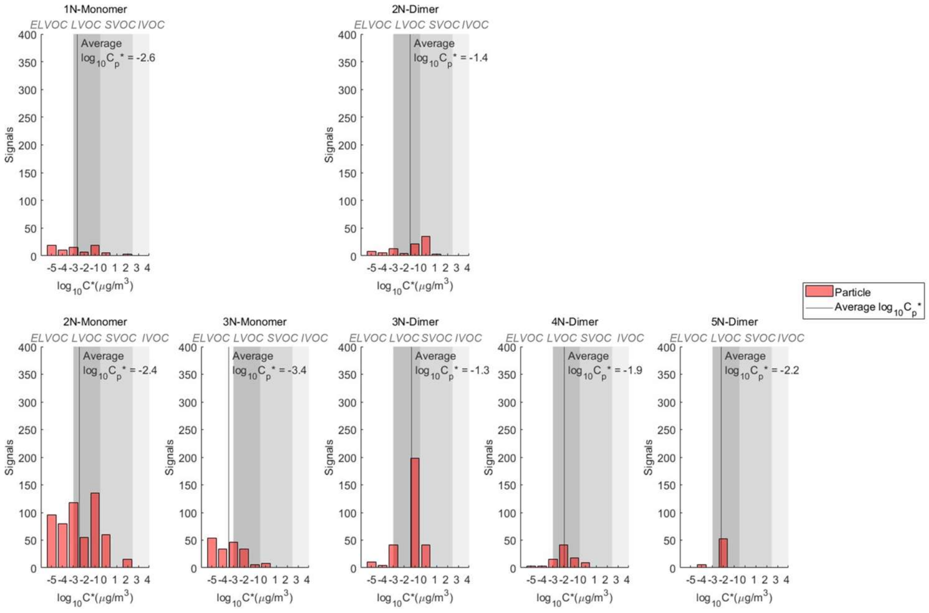

Figure S6. Particle-phase product only volatility distributions calculated using (a) the partitioning method and (b) the thermogram method (experiment B4). Each panel represents a different chemical family. The first row represents first-generation products. The second row represents second-generation products. Each shade of grey represents IVOC, SVOC, LVOC, ELVOC, respectively

(a)

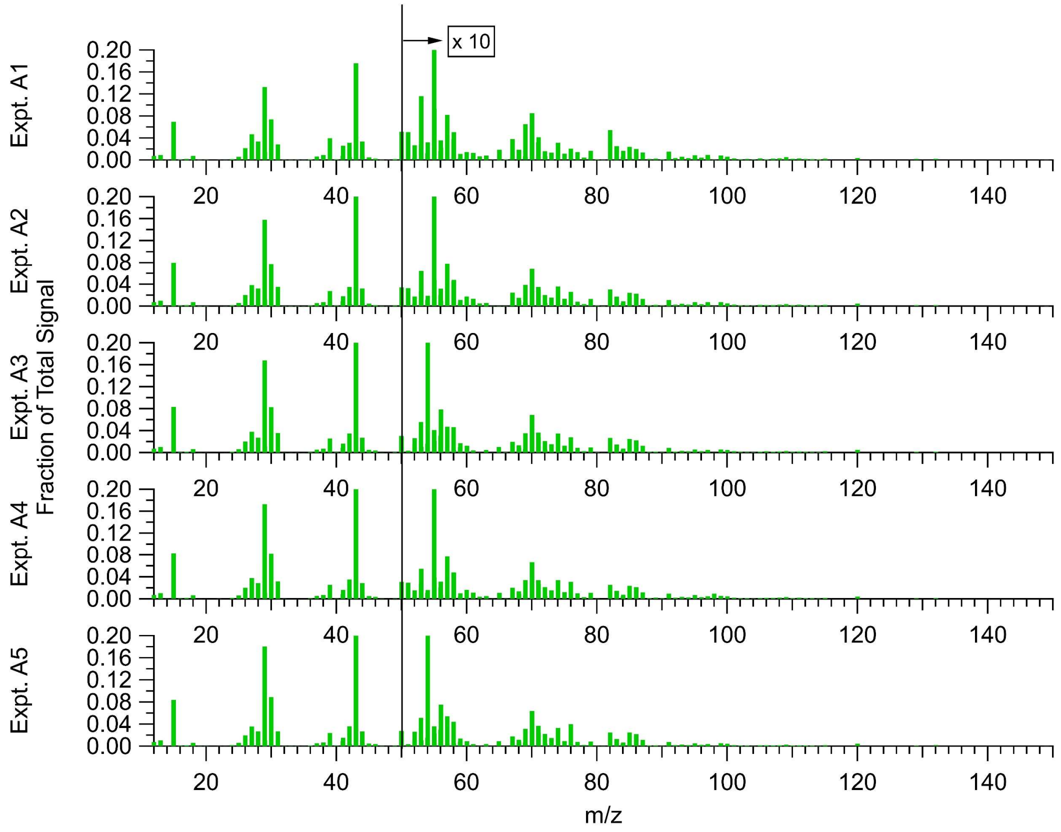
x 10
Expt. A1
Expt. A2
Expt. A3
Expt. A4
Expt. A5
Fraction of Total Signal
0.20
0.16
0.12
0.08
0.04
0.00
20
40
60
80
100
120
140
m/z

(b)

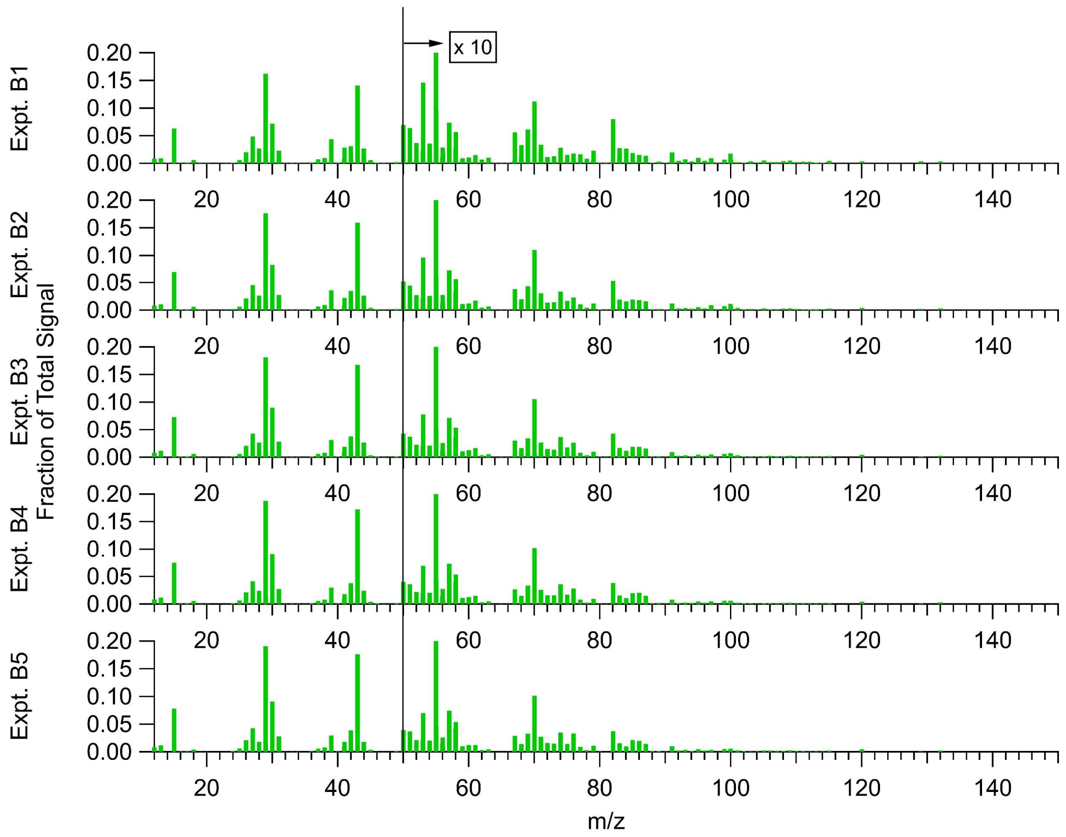

Figure S7. Normalized AMS mass spectra of (a) experiments A1-A5 and (b) experiments B1-B5.

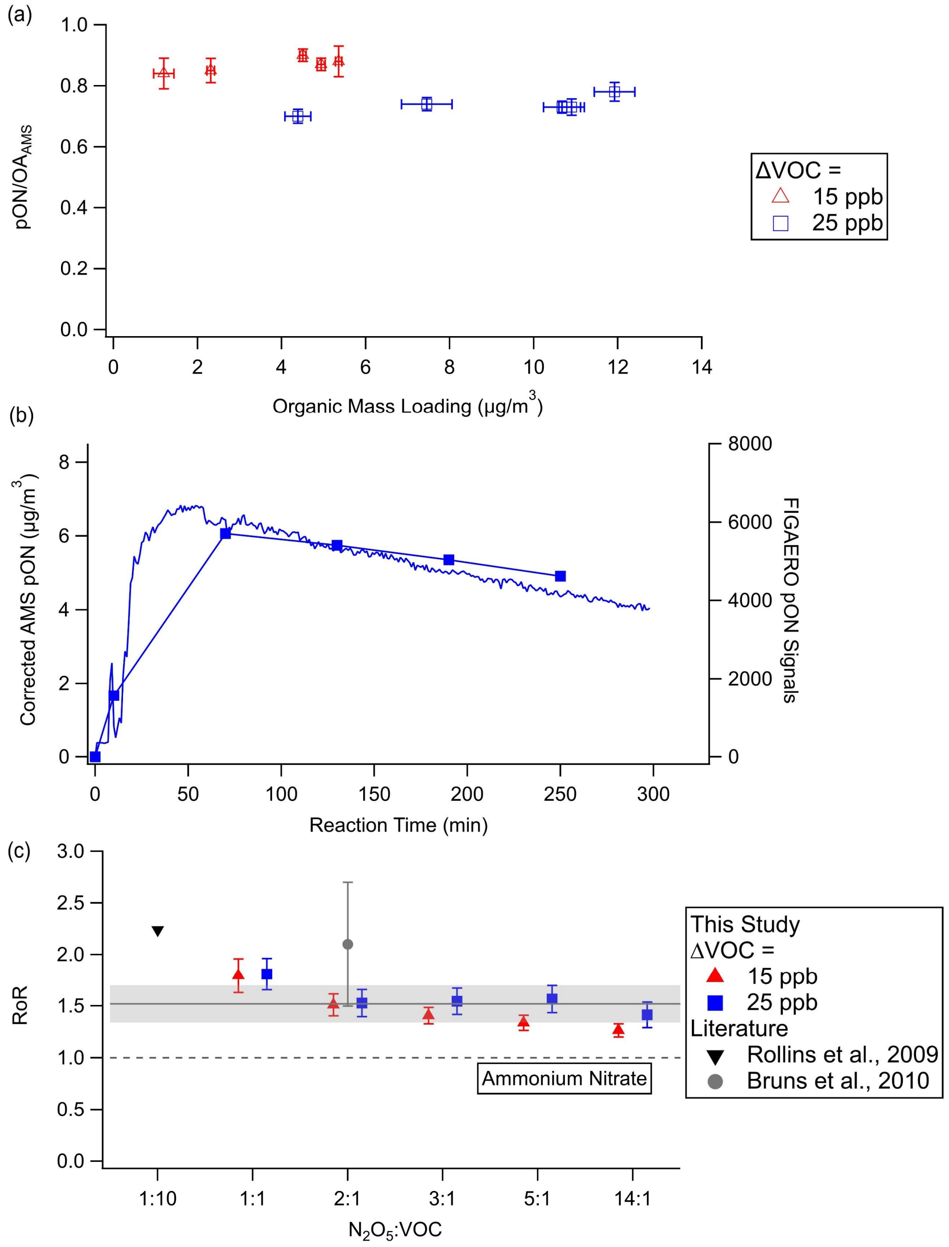

Figure S8. (a) Particulate organic nitrates normalized by total organic aerosol values (pON/OA) in this study, (b) Comparison of pON measured in FIGAERO-CIMS vs. AMS in experiment B4, the AMS data has been corrected by collection efficiency (CE, by comparison of AMS and SMPS measurements) (c) RoR values in this study compared against literature values in Rollins et al and Bruns et al.[10, 11] For the individual RoR values in this study, the uncertainties (1σ) are from scatter in the NO_2^+/NO^+ ratio (NO_x ratio) at the peak SOA period. The solid grey line and shaded area are the average value and corresponding uncertainty (1σ) of all our RoR data points, respectively. It is noted that the N_2O_5:VOC ratios for Rollins et al. and Bruns et al. are calculated based on the N_2O_5 and VOC concentrations reported in their studies.[10, 11] No uncertainty was reported in Rollins et al.[10]

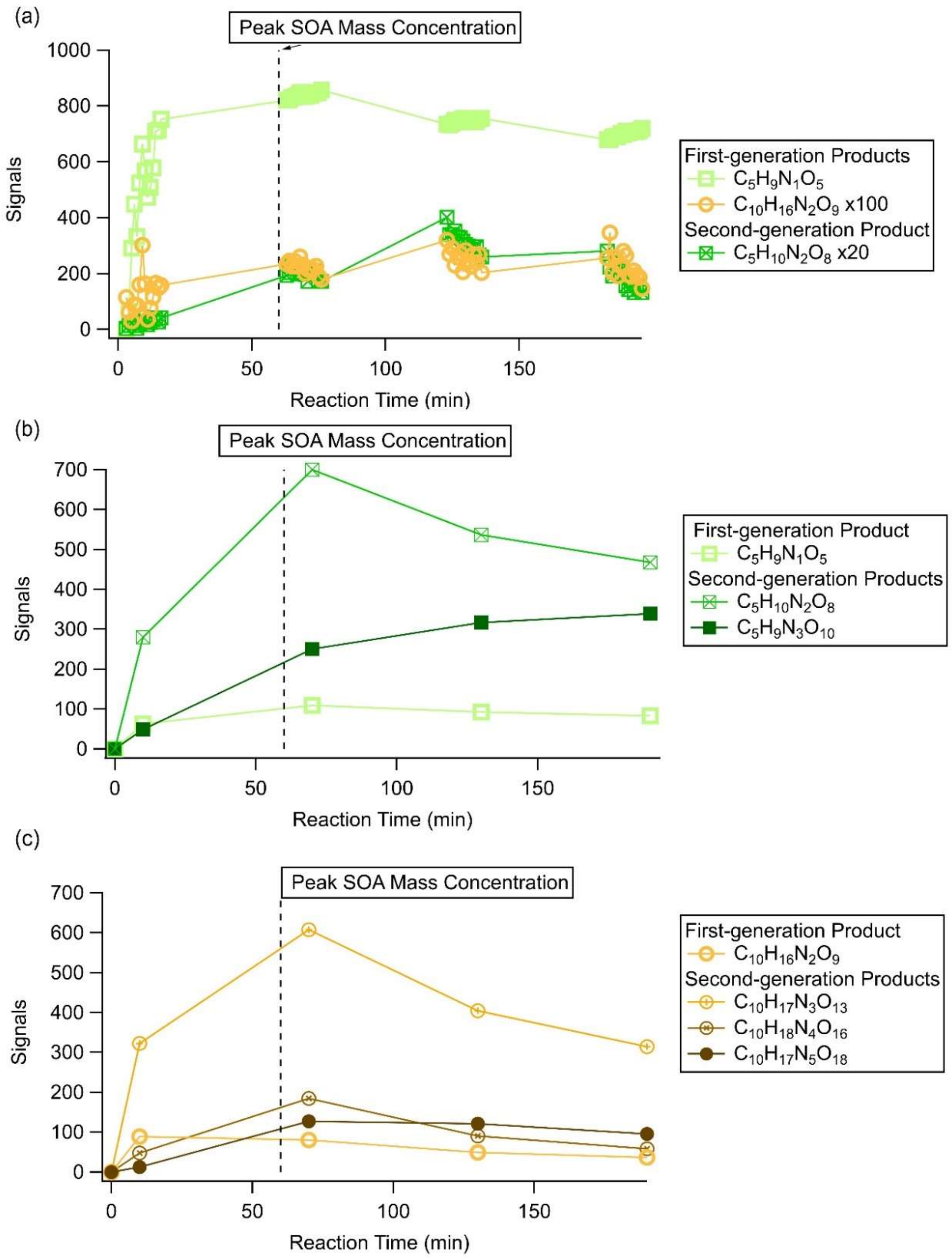

Figure S9. Example time series of signature compounds in each product family in (a) gas phase, (b) particle phase (monomers), and (c) particle phase (dimers) in experiment A4. (not wall loss corrected). Most of the second-generation products tend to have lower volatility to facilitate partitioning into particle phase. Therefore, their signals are low in gas phase without clear trends.

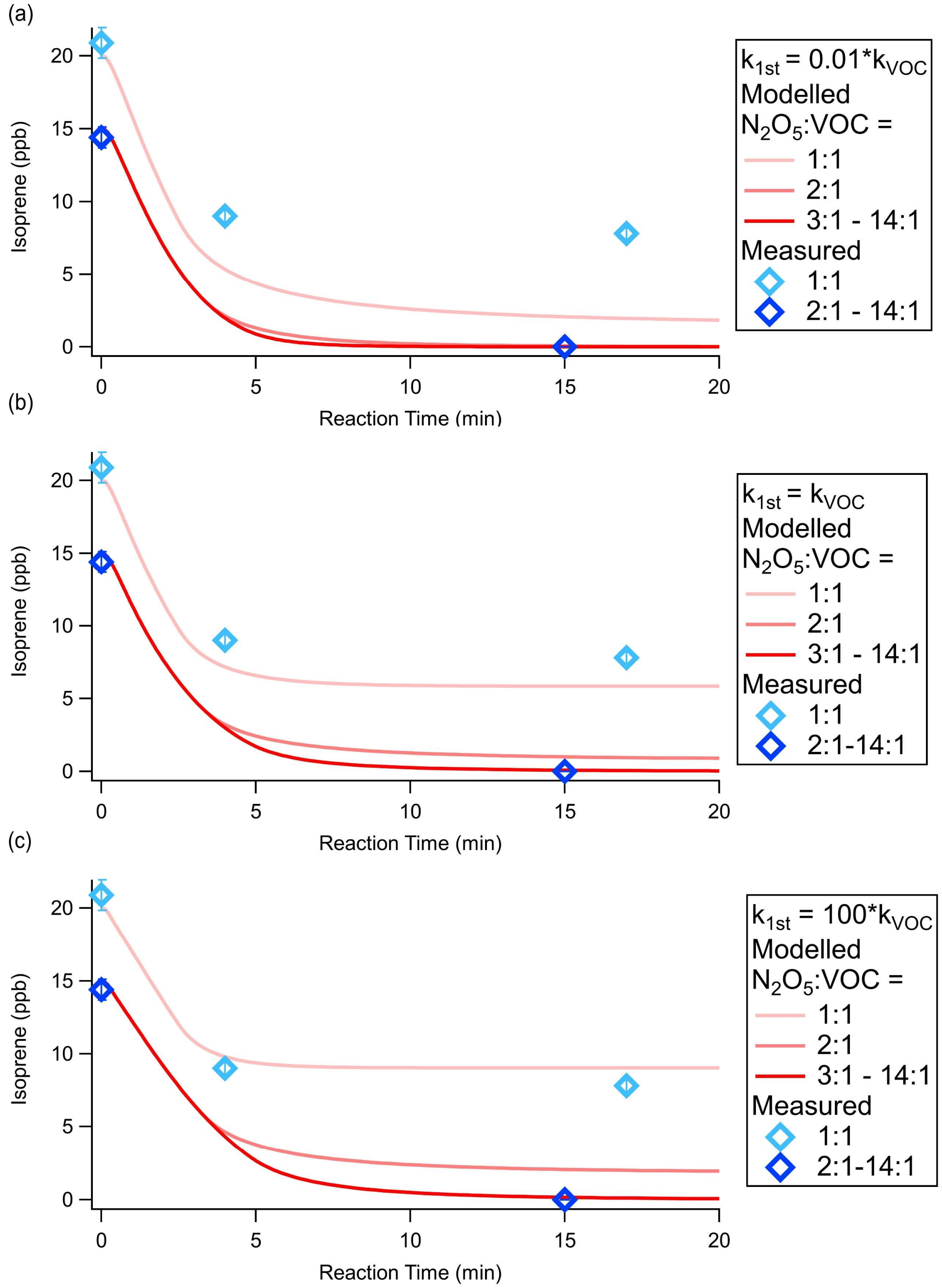

Figure S10. Time series of modeled isoprene decay under the assumption of: (a) k_{1st} = $0.01*k_{VOC}$, (b) $k_{1st} = k_{VOC}$, (c) $k_{1st} = 100*k_{VOC}$.

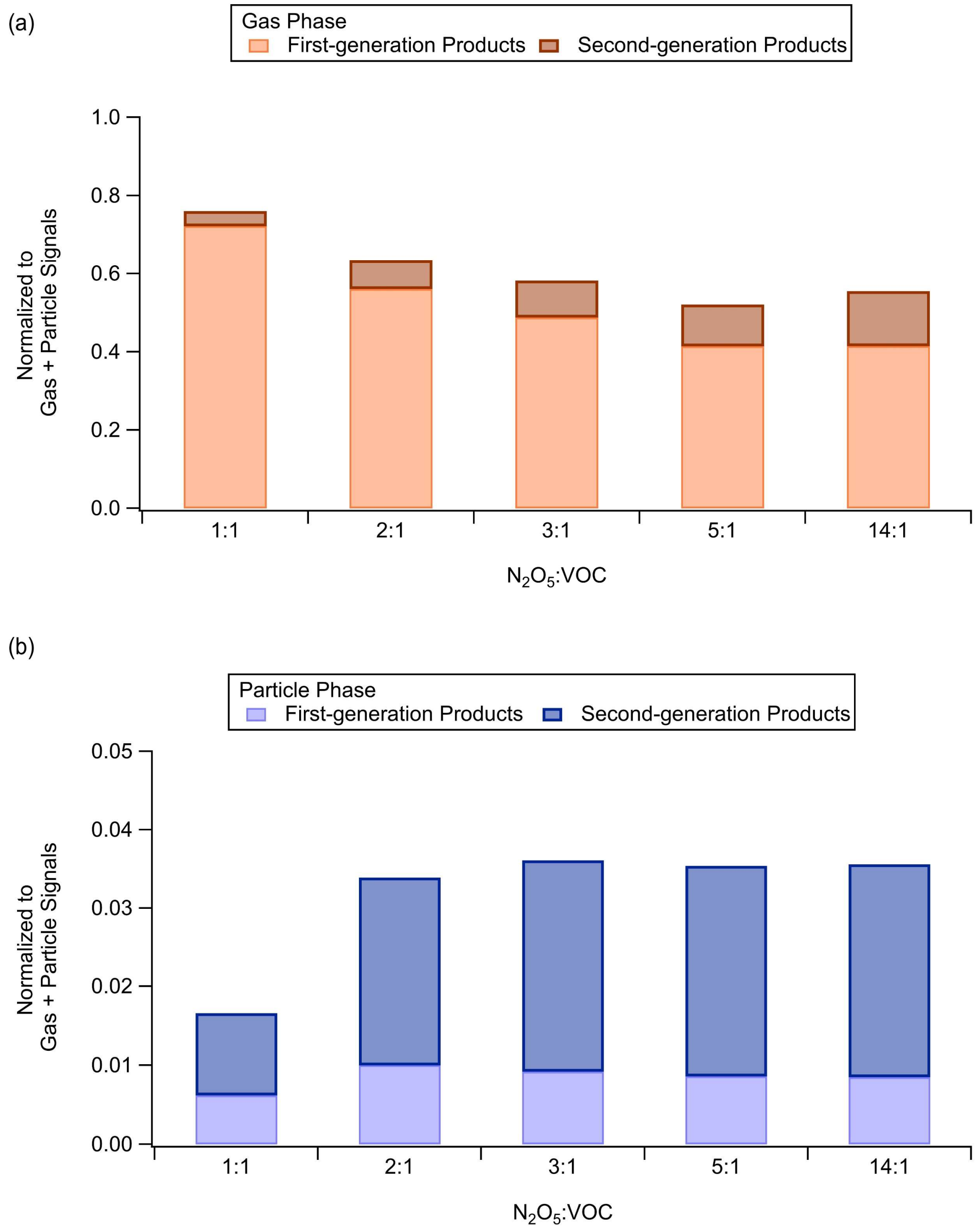

Figure S11. Distribution of bulk first- vs. second-generation products in gas and particle phases for experiment A1-A5, normalized by total organics in both phases.

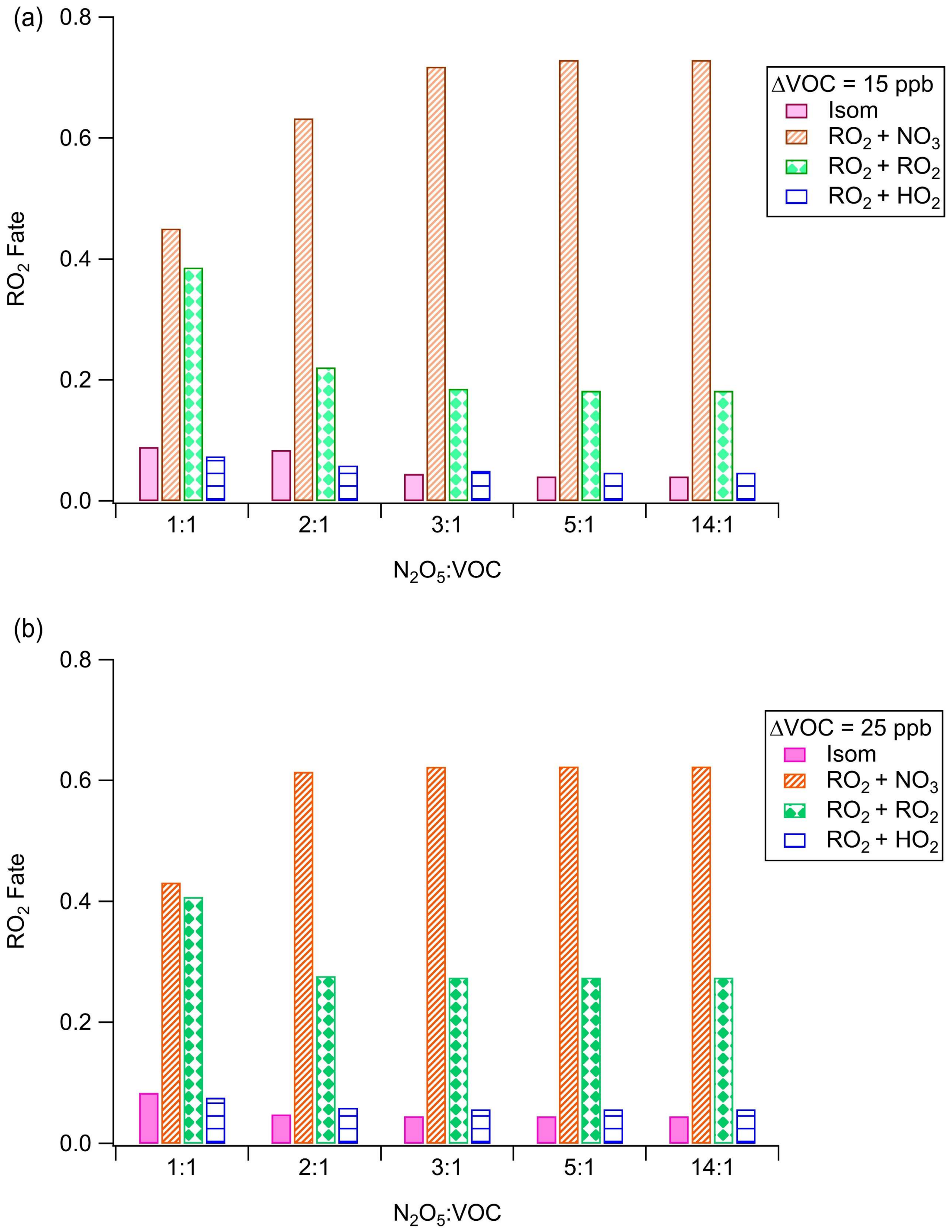

Figure S12. 0-D model results on reaction pathways of first-generation RO_2 for (a) 15 ppb isoprene and (b) 25 ppb isoprene. The input kinetic data are obtained from Vereecken et al., 2021.[5]

Table S1. Summary of R_{AN} and R_{ON} applied to calculate the mass concentration of particulate organic nitrates. R_{AN} from 300 nm ammonium nitrate particles on the nearest IE calibration date to experiments is used unless noted otherwise.

Set	Expt	R_{AN}	R_{ON}	RoR
**A**	1	1.88	3.53	1.81
	2		2.88	1.53
	3		2.91	1.55
	4		2.95	1.57
	5		2.65	1.41
**B**	1	3.08	5.51	1.79
	2		4.65	1.51
	3		4.34	1.41
	4		4.13	1.34
	5		3.91	1.27

Table S2. Summary of sensitivity of particulate organic nitrates under all conditions.

Expt	pON sensitivity, FIGAERO to AMS corrected by collection efficiency (Hz/ppt)
A1	7.33 ± 0.68
A2	5.78 ± 0.48
A3	5.26 ± 0.41
A4	5.18 ± 0.35
A5	5.34 ± 0.41
B1	7.70 ± 0.76
B2	7.90 ± 0.68
B3	8.64 ± 0.78
B4	8.91 ± 0.63
B5	7.51 ± 0.75

Table S3. Fitted parameters for yield curves in Figure 6.

**Study**	α_1	$\boldsymbol{\alpha_2}$	$\mathbf{K_{om,1}}$	$\mathbf{K_{om,2}}$
Ng et al., 2008	0.089	0.203	0.182	0.046
This Study: N_2O_5:VOC = 1:1	0.038	0.176	1.287	0.037
This Study: N_2O_5:VOC = 2:1	0.110	0.068	0.202	0.197
This Study: N_2O_5:VOC = 3:1 – 14:1	0.115	0.125	0.400	0.096

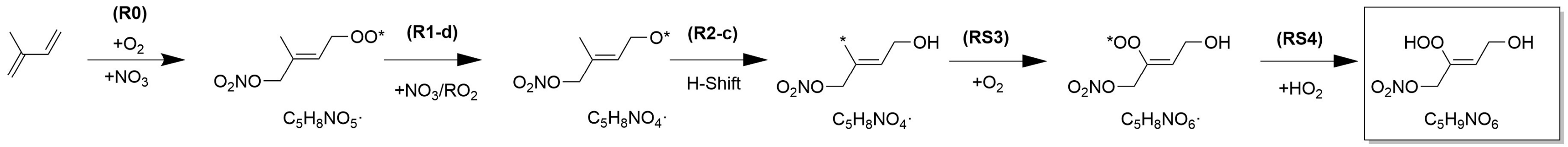

Scheme S1. An example of proposed formation mechanism of $C_5H_9NO_6$ with autoxidation pathways, which can form compounds with the same carbon, hydrogen, nitrogen atom numbers as those in Scheme 1 but with different oxygen number.

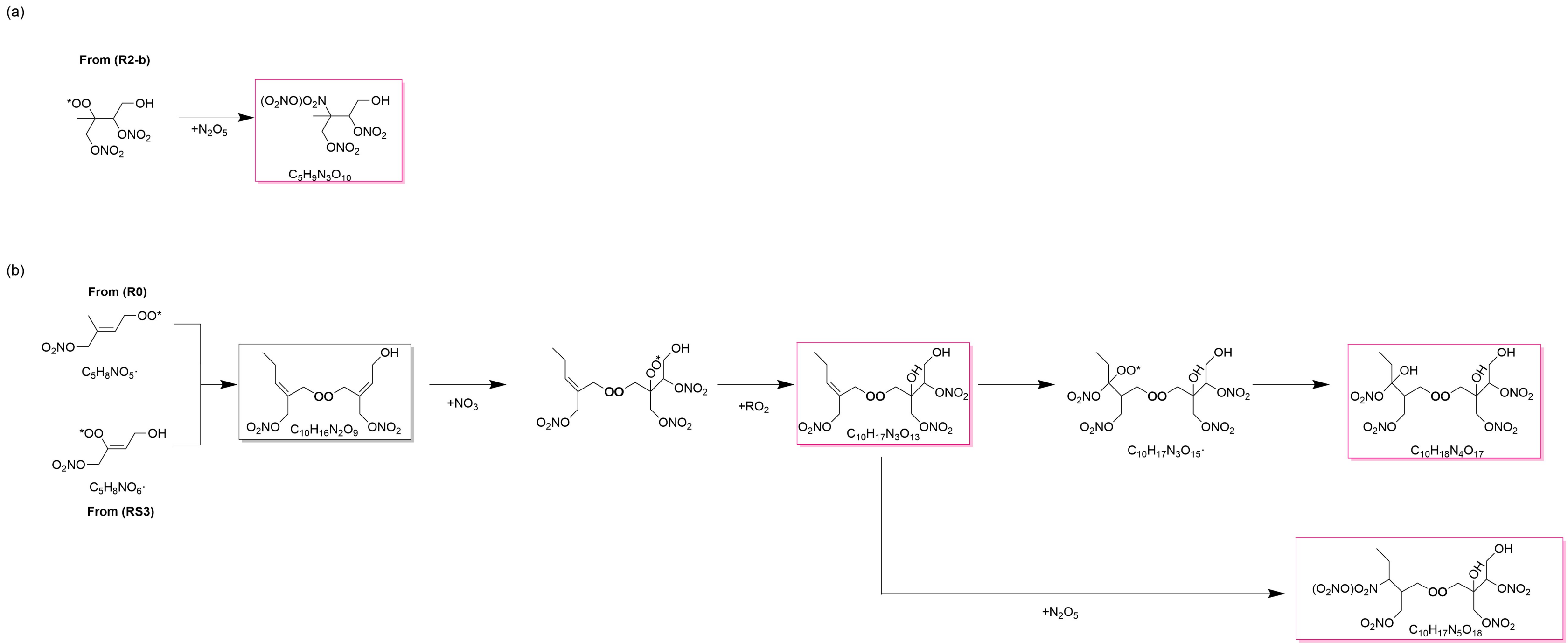

Scheme S2. Proposed formation mechanism of $C_5H_9N_3O_{10}$ and $C_{10}H_{17}N_5O_{18}$ with direct addition of N_2O_5 to C=C double bond.

References

1. Wolfe, G. M.; Marvin, M. R.; Roberts, S. J.; Travis, K. R.; Liao, J., The Framework for 0-D Atmospheric Modeling (F0AM) v3.1. *Geosci. Model Dev.* **2016,** *9* (9), 3309-3319.
2. Jenkin, M. E.; Young, J. C.; Rickard, A. R., The MCM v3.3.1 degradation scheme for isoprene. *Atmos. Chem. Phys.* **2015,** *15* (20), 11433-11459.
3. Schwantes, R. H.; Teng, A. P.; Nguyen, T. B.; Coggon, M. M.; Crounse, J. D.; St. Clair, J. M.; Zhang, X.; Schilling, K. A.; Seinfeld, J. H.; Wennberg, P. O., Isoprene NO3 Oxidation Products from the RO2 + HO2 Pathway. *The Journal of Physical Chemistry A* **2015,** *119* (40), 10158-10171.
4. Wennberg, P. O.; Bates, K. H.; Crounse, J. D.; Dodson, L. G.; McVay, R. C.; Mertens, L. A.; Nguyen, T. B.; Praske, E.; Schwantes, R. H.; Smarte, M. D.; St Clair, J. M.; Teng, A. P.; Zhang, X.; Seinfeld, J. H., Gas-Phase Reactions of Isoprene and Its Major Oxidation Products. *Chemical Reviews* **2018,** *118* (7), 3337-3390.
5. Vereecken, L.; Carlsson, P. T. M.; Novelli, A.; Bernard, F.; Brown, S. S.; Cho, C.; Crowley, J. N.; Fuchs, H.; Mellouki, W.; Reimer, D.; Shenolikar, J.; Tillmann, R.; Zhou, L.; Kiendler-Scharr, A.; Wahner, A., Theoretical and experimental study of peroxy and alkoxy radicals in the NO3-initiated oxidation of isoprene. *Physical Chemistry Chemical Physics* **2021,** *23* (9), 5496-5515.
6. Dewald, P.; Liebmann, J. M.; Friedrich, N.; Shenolikar, J.; Schuladen, J.; Rohrer, F.; Reimer, D.; Tillmann, R.; Novelli, A.; Cho, C.; Xu, K.; Holzinger, R.; Bernard, F.; Zhou, L.; Mellouki, W.; Brown, S. S.; Fuchs, H.; Lelieveld, J.; Crowley, J. N., Evolution of NO3 reactivity during the oxidation of isoprene. *Atmos. Chem. Phys.* **2020,** *20* (17), 10459-10475.
7. Farmer, D. K.; Matsunaga, A.; Docherty, K. S.; Surratt, J. D.; Seinfeld, J. H.; Ziemann, P. J.; Jimenez, J. L., Response of an aerosol mass spectrometer to organonitrates and organosulfates and implications for atmospheric chemistry. *Proceedings of the National Academy of Sciences* **2010,** *107* (15), 6670-6675.
8. Ng, N. L.; Brown, S. S.; Archibald, A. T.; Atlas, E.; Cohen, R. C.; Crowley, J. N.; Day, D. A.; Donahue, N. M.; Fry, J. L.; Fuchs, H.; Griffin, R. J.; Guzman, M. I.; Herrmann, H.; Hodzic, A.; Iinuma, Y.; Jimenez, J. L.; Kiendler-Scharr, A.; Lee, B. H.; Luecken, D. J.; Mao, J.; McLaren, R.; Mutzel, A.; Osthoff, H. D.; Ouyang, B.; Picquet-Varrault, B.; Platt, U.; Pye, H. O. T.; Rudich, Y.; Schwantes, R. H.; Shiraiwa, M.; Stutz, J.; Thornton, J. A.; Tilgner, A.; Williams, B. J.; Zaveri, R. A., Nitrate radicals and biogenic volatile organic compounds: oxidation, mechanisms, and organic aerosol. *Atmos. Chem. Phys.* **2017,** *17* (3), 2103-2162.
9. Fry, J. L.; Draper, D. C.; Zarzana, K. J.; Campuzano-Jost, P.; Day, D. A.; Jimenez, J. L.; Brown, S. S.; Cohen, R. C.; Kaser, L.; Hansel, A.; Cappellin, L.; Karl, T.; Hodzic Roux, A.; Turnipseed, A.; Cantrell, C.; Lefer, B. L.; Grossberg, N., Observations of gas- and aerosol-phase organic nitrates at BEACHON-RoMBAS 2011. *Atmos. Chem. Phys.* **2013,** *13* (17), 8585-8605.
10. Rollins, A. W.; Kiendler-Scharr, A.; Fry, J. L.; Brauers, T.; Brown, S. S.; Dorn, H. P.; Dubé, W. P.; Fuchs, H.; Mensah, A.; Mentel, T. F.; Rohrer, F.; Tillmann, R.; Wegener, R.; Wooldridge, P. J.; Cohen, R. C., Isoprene oxidation by nitrate radical:

alkyl nitrate and secondary organic aerosol yields. *Atmos. Chem. Phys.* **2009,** *9* (18), 6685-6703.
11. Bruns, E. A.; Perraud, V.; Zelenyuk, A.; Ezell, M. J.; Johnson, S. N.; Yu, Y.; Imre, D.; Finlayson-Pitts, B. J.; Alexander, M. L., Comparison of FTIR and Particle Mass Spectrometry for the Measurement of Particulate Organic Nitrates. *Environmental Science & Technology* **2010,** *44* (3), 1056-1061.
12. Day, D. A.; Campuzano-Jost, P.; Nault, B. A.; Palm, B. B.; Hu, W.; Guo, H.; Wooldridge, P. J.; Cohen, R. C.; Docherty, K. S.; Huffman, J. A.; de Sá, S. S.; Martin, S. T.; Jimenez, J. L., A systematic re-evaluation of methods for quantification of bulk particle-phase organic nitrates using real-time aerosol mass spectrometry. *Atmos. Meas. Tech.* **2022,** *15* (2), 459-483.
13. Takeuchi, M.; Ng, N. L., Chemical composition and hydrolysis of organic nitrate aerosol formed from hydroxyl and nitrate radical oxidation of α-pinene and β-pinene. *Atmos. Chem. Phys.* **2019,** *19* (19), 12749-12766.
14. Takeuchi, M.; Wang, Y.; Nault, B. A.; Chen, Y.; Canagaratna, M. R.; Ng, N. L., Evaluating the response of the Aerodyne aerosol mass spectrometer to monoterpene- and isoprene-derived organic nitrate standards. *Aerosol Science and Technology* **2024**, 1-18.
15. Robinson, M. A.; Roberts, J. M.; Neuman, J. A.; Jernigan, C. M.; Xu, L.; Coggon, M. M.; Stockwell, C. E.; Warneke, C.; Peischl, J.; Gilman, J. B.; Lamplugh, A.; Rollins, A. W.; Zuraski, K.; Rivera-Rios, J. C.; Wang, Y.; Ng, N. L.; Liu, S.; Brown, S. S.; Veres, P. R., Online Calibration of a Chemical Ionization Mass Spectrometer for Multifunctional Biogenic Organic Nitrates. *ACS ES&T Air* **2024**.
16. Lopez-Hilfiker, F. D.; Mohr, C.; Ehn, M.; Rubach, F.; Kleist, E.; Wildt, J.; Mentel, T. F.; Carrasquillo, A. J.; Daumit, K. E.; Hunter, J. F.; Kroll, J. H.; Worsnop, D. R.; Thornton, J. A., Phase partitioning and volatility of secondary organic aerosol components formed from α-pinene ozonolysis and OH oxidation: the importance of accretion products and other low volatility compounds. *Atmos. Chem. Phys.* **2015,** *15* (14), 7765-7776.
